# The genomic and functional landscapes of developmental plasticity in the American cockroach

**DOI:** 10.1038/s41467-018-03281-1

**Published:** 2018-03-20

**Authors:** Sheng Li, Shiming Zhu, Qiangqiang Jia, Dongwei Yuan, Chonghua Ren, Kang Li, Suning Liu, Yingying Cui, Haigang Zhao, Yanghui Cao, Gangqi Fang, Daqi Li, Xiaoming Zhao, Jianzhen Zhang, Qiaoyun Yue, Yongliang Fan, Xiaoqiang Yu, Qili Feng, Shuai Zhan

**Affiliations:** 1 Guangzhou Key Laboratory of Insect Development Regulation and Application Research, Institute of Insect Science and Technology & School of Life Sciences, South China Normal University, Guangzhou, 510631 China; 2 CAS Key Laboratory of Insect Developmental and Evolutionary Biology, CAS Center for Excellence in Molecular Plant Sciences, Institute of Plant Physiology and Ecology, Chinese Academy of Sciences, Shanghai, 200032 China; 3 University of Chinese Academy of Sciences, Beijing, 100049 China; 4 Research Institute of Applied Biology, Shanxi University, Taiyuan, 030006 China; 5 Zhongshan Entry-Exit Inspection and Quarantine Bureau Technology Center, Zhongshan, 528403 China; 6 State Key Laboratory of Crop Stress Biology for Arid Areas and Key Laboratory of Integrated Pest Management on the Loess Plateau of Ministry of Agriculture, Northwest A&F University, Yangling, 712100 China

## Abstract

Many cockroach species have adapted to urban environments, and some have been serious pests of public health in the tropics and subtropics. Here, we present the 3.38-Gb genome and a consensus gene set of the American cockroach, *Periplaneta americana*. We report insights from both genomic and functional investigations into the underlying basis of its adaptation to urban environments and developmental plasticity. In comparison with other insects, expansions of gene families in *P. americana* exist for most core gene families likely associated with environmental adaptation, such as chemoreception and detoxification. Multiple pathways regulating metamorphic development are well conserved, and RNAi experiments inform on key roles of 20-hydroxyecdysone, juvenile hormone, insulin, and decapentaplegic signals in regulating plasticity. Our analyses reveal a high level of sequence identity in genes between the American cockroach and two termite species, advancing it as a valuable model to study the evolutionary relationships between cockroaches and termites.

## Abstract (web summary)

The American cockroach (*Periplaneta americana*) is an hemimetabolous insect with rapid growth, high fecundity, and remarkable tissue-regeneration capability. Here Li et al sequence its 3.38-Gb genome and perform the functional studies, yielding insights into its environmental adaptation and developmental plasticity.

## Introduction

Many species of cockroaches (Insecta, Blattodea) are serious cosmopolitan pests of public health. The American cockroach, *Periplaneta americana* (Blattodae), is one of the largest insect species that lives in close proximity with humans (Supplementary Fig. 1). Despite its common name, the American cockroach was introduced to the United States of America from Africa in the early 16^th^ century and has since spread throughout the world; it is famous for its household name “Xiao Qiang” (a small pest with strong living vitality) in China and “Waterbug” in America. The American cockroach prefers indoor environments with access to food sources, but can be found outdoors in moist, shady, and warm areas. It consumes a great variety of substrates, with a particular preference for fermenting foods^1^. It also transfers disease-causing organisms such as bacteria, protozoa, and viruses; and triggers allergic reactions and asthma in certain individuals^2^. The American cockroach is thus a serious urban pest in both warm and humid regions. Beyond serving as a pest, this cockroach is also important in traditional Chinese medicine, well documented in Chinese medical encyclopedia, such as *Ben Cao Gang Mu* and *Shen Nong Ben Cao Jing*. Moreover, its ethanol extract has been developed as a prescribed drug (*Kang Fu Xin Ye*) for wound healing and tissue repair (National Drug Standard WS_3_-B-3674-2000(Z)).

The American cockroach provides a potential model to study the biology of a hemimetabolous insect with rapid growth, metamorphosis after variable molts (6-14), high fecundity, and remarkable tissue regeneration capability^1^. Here, we report the genomic resources of three common *Periplaneta* species focusing on the genome assembly, consensus gene set, and evolutionary analyses of *P. americana*. We also present the functional studies and analyses of gene families and signaling pathways likely involved in major aspects of environmental adaptation and developmental plasticity in the American cockroach (Supplementary Fig. 2).

## Results and Discussion

### Genome assembly and annotation

We sequenced three members of the genus *Periplaneta* and generated >1 Tb of data (Supplementary Table 1). For de novo assembly, we sequenced *P. americana* to 295 × fold coverage (Supplementary Table 1). Two other sibling species, *P. australasiae* (the Australian cockroach) and *P. fuliginosa* (the smokybrown cockroach), were sequenced to approximately 40 × fold coverage for genome comparisons (Supplementary Table 1). The assembly of *P. americana* yielded 3.38 Gb of reference genomic sequence, with an N_50_ length of 333 kb (Supplementary Table 2). The assembled size is consistent with a previous estimation by flow cytometry^3^. We assessed the completeness and quality of the assembly using both its transcripts and conserved genes from a variety of resources (Supplementary Table 2 and 3). Since these sequences of all independent resources could be recovered at a high coverage (98–100%; Supplementary Table 3), the assembly is suggested to be a high-quality representation of the *P. americana* genome.

In comparison with the sequenced genomes of other insect species, the American cockroach genome is the second largest after that of the locust, *Locusta migratoria*^4^. Similar to the genome of *L. migratoria*, approximately 60% of the genome of *P. americana* is composed of repetitive elements (Supplementary Table 2), supporting the hypothesis that repetitive elements drive the evolution of genome sizes^4^. Other genomic features of *P. americana*, such as the percentages of GC content and repetitive elements, are typical of Blattodea (Supplementary Table 2).

An official gene set was generated by integrating ab initio predictions, alignments of insect gene homologs, and evidence of full-length *P. americana* transcriptomes. In the first version of the gene set (OGS1.0), we reported 21,336 protein-coding genes, 95% of which were detected to be expressed (Supplementary Table 4). Assessment of completeness revealed the gene set is also of high quality (Supplementary Table 4). Overall, *P. americana* encodes a typical blattodean gene repertoire, of which 90% genes match entries of Blattodea homologs, and 84% and 82% have homologs in another cockroach species (the German cockroach, *Blattella germanica*^5^) and a termite species (the dampwood termite, *Zootermopsis nevadensis*^6^), respectively (Supplementary Table 5). Features of the *P. americana* genome, such as the exon number per gene or transcript length, are comparable to those of other insects, except for intron length (Supplementary Table 4). The median length of introns predicted from the *P. americana* genome is 3 Kb, which is half of that of *L. migratoria* and greater than those of other sequenced insects. This finding supports the positive correlation between intron size and genome size as previously described^4^.

### Genome evolution in Blattodea

**Fig. 1:**
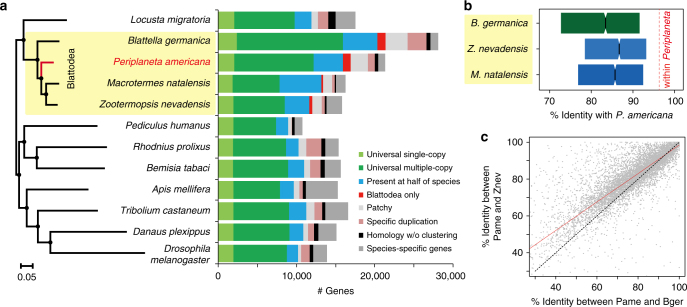
Orthology and genome evolution of Blattodea. **a** Orthology assignment of four blattodean species and eight other representative insect species (Supplementary Table 6). Bars are subdivided to represent different types of orthology clusters as indicated. Universal groups represent common gene families across all species analyzed, but absence in at most one genome is tolerated; of them, “single-copy” tolerates absence or duplication in a single genome, while “multiple-copy” indicates other universal genes. “Blattodea only” indicates unique presence to the blattodean lineage and in at least three genomes. “Specific” groups indicate specific presence or duplication in only one species. “Homology” indicates partial homology detected with E < 10^−5^ but no orthology grouped. Remaining orthologs were assigned to “Present at half of species” or “Patchy”, depending on whether presence is in at least six genomes or not. The phylogeny was calculated using maximum-likelihood analyses of a concatenated alignment of 538 exactly single-copy proteins. The tree was rooted using the sister clades of Blattodea and Orthoptera species as described previously^70^. Bootstrap values based on 100 replicates are equal to 100 for each node. **b** Distribution of sequence identities. The boxplots delineate the interval between the first and the third quartiles of the identity distribution between *P. americana* and one indicated blattodean species. Notch indicates the median value. Amino acid sequence identity was calculated based on multiple alignment of each universal single-copy ortholog (5911 in total) in four blattodean species. The red dashed line indicates the median value within *Periplaneta* species (96%). **c** Comparison of sequence identities on each ortholog cluster. A total of 7640 1:1:1 orthologs were identified among *P. americana*, *B. germanica*, and *Z. nevadensis*. Each dot represents such an ortholog. Value on *x*-axis indicates the sequence identity between *P. americana* (Pame) and *B. germanica* (Bger), while value on *y*-axis indicates that between *P. americana* and *Z. nevadensis* (Znev). Red line indicates the smoother of a locally weighted regression, with the coefficient of determination as 0.99999. Dashed line indicates positions where the sequence identity between *P. americana* and *B. germanica* is identical to that between *P. americana* and *Z. nevadensis*

We compared the gene repertoires of 12 representative insect species (Fig. 1a; Supplementary Table 6), including three sequenced blattodean species: (1) the German cockroach, *B. germanica* (Ectobiidae)^5^; (2) the dampwood termite, *Z. nevadensis* (Termopsidae)^6^; and (3) the fungus growing termite, *Macrotermes natalensis* (Termitidae)^7^. We identified 479 Blattodea-specific orthologs, representing approximately 1000 genes in each gene set of the two cockroaches, *P. americana* and *B. germanica*. These two cockroach genomes encode the most genes across the species we analyzed. Unlike the pea aphid (not analyzed here)^8^, which encodes the largest number of genes in insects investigated to date, these cockroach genomes encode much fewer species-specific genes (Fig. 1a). In contrast, the increase in gene number in cockroaches is mainly attributed to their expansion of universal genes in the 12 species we analyzed (Supplementary Table 7). The gene sets of *P. americana* and *B. germanica* contained 13,555 and 10,107 multicopy universal genes, respectively, while the highest number of such genes in other insects was only 7708 (*L. migratoria*) (Fig. 1a).

We also identified approximately 2000 single-copy universal genes for each species and selected 538 out of them to reconstruct the phylogeny (Fig. 1a). The analysis showed the lineage of Blattodea (including Isoptera)^9^ is monophyletic. In this lineage, the American cockroach has a closer relationship with the two termites than the German cockroach. We then focused comparative analyses on gene families in six available Blattodea genomes (see Methods). Compared with *P. americana*, the two sibling species, *P. fuliginosa* and *P. australasiae*, presented approximately 88% amino acid identity to orthologous proteins. Notably, we found that *P. americana* shares only 75% sequence identity with *B. germanica*, which is lower than that with the two genome-sequenced termites, i.e., 79% to *Z. nevadensis* and 80% to *M. natalensis*. To exclude potential biases, we further limited the comparisons within the common aligned blocks across all analyzed species, showing an overall higher level of sequence identities between the American cockroach and the two termites (Fig. 1b). This finding suggests that the American cockroach is more closely related to the termites, at least the two we analyzed, than to the German cockroach. We found the German cockroach and the termite (*Z. nevadensis*) share 9633 and 9573 orthologs with the American cockroach, respectively. Of the 7640 common orthologs, we found approximately two-thirds of the American cockroach genes are more closely related to the termite in sequence identity, while only one-third are more closely related to the German cockroach, strengthening the above finding (Fig. 1c). Genes conserved more between the American cockroach and the termites were found significantly over-represented in 29 pathways, including a number of classic functional components in insects, such as development, nutrition, and immunity (Supplementary Table 8). The high level of sequence identity between the American cockroach and the termites provides a solid relationship between cockroaches and termites and enhances the evolutionary significance of *P. americana* by filling a gap during the evolution of available blattodean resources. Instead, genes more conserved between the American cockroach and the German cockroach were significantly enriched in only six pathways. Interestingly, two of them are related to signal transduction (Supplementary Table 9), suggesting a similar role in processing environmental information between the two common cockroaches.

### Environmental adaptation

**Fig. 2:**
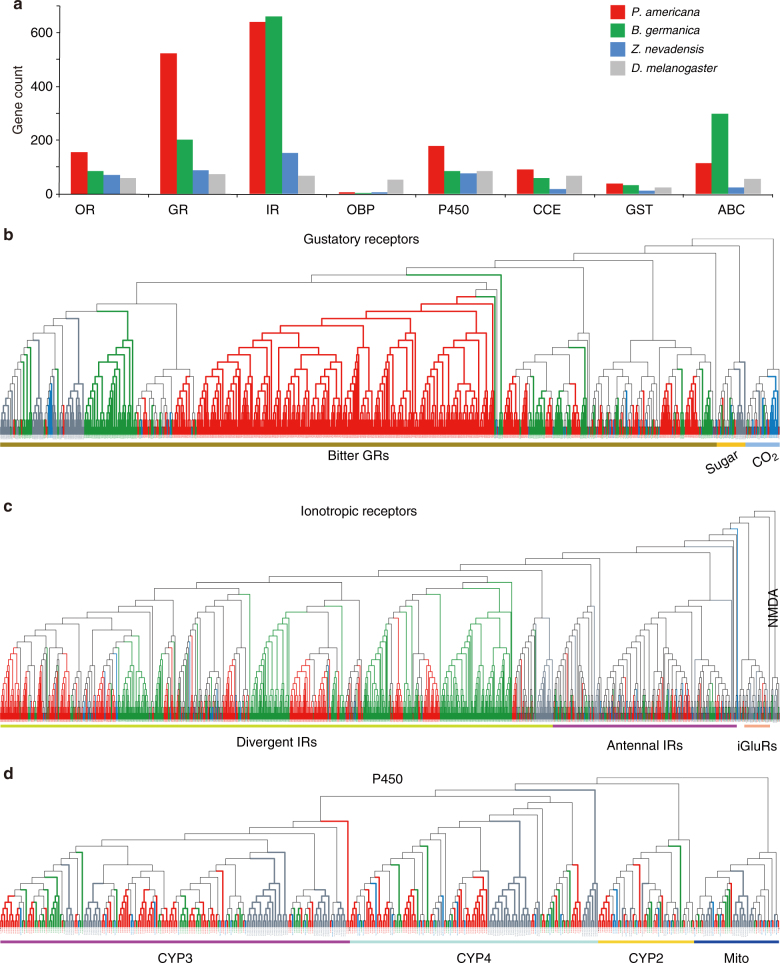
Gene families involved in chemoreception and detoxification in *P. americana* and other blattodean species. **a** Counts of chemosensory- and detoxification-related gene families in the genomes of three blattodean species and *Drosophila melanogaster*. OR olfactory receptor, GR gustatory receptor, IR ionotropic receptor, OBP odorant-binding protein, P450 cytochromes P450 (CYP), CCE carboxyl/choline esterases, GST glutathione *S*-transferase, ABC ATP-binding cassette. Of these, three gene families with massive expansions in *P. americana* were selected for maximum-likelihood phylogenetic analysis, as shown in **b**–**d**, representing GRs, IRs, and P450, respectively. Phylogenetic relationships of other gene families are shown in Supplementary Figs. 3–7. iGluRs ionotropic glutamate receptors, NMDA *N*-methyl-d-aspartate receptors, Mito the mitochondrial clan

**Fig. 3:**
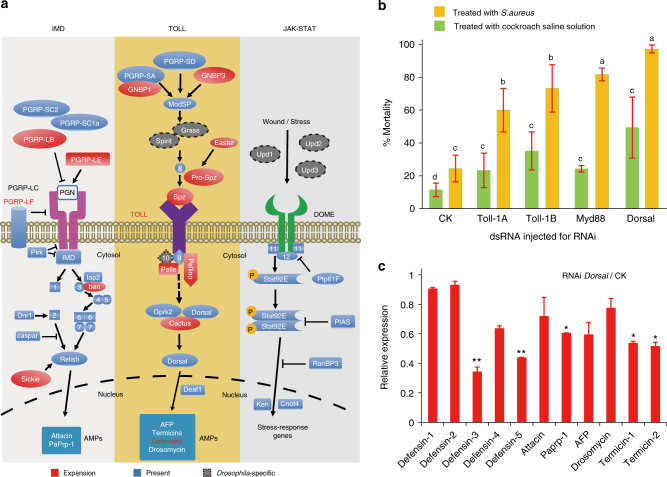
Gene repertoire of the innate immune system and functional analyses of the Toll pathway in *P. americana*. **a** Representation of three main innate immune signaling pathways (Imd, Toll, and JAK-STAT, proposed in *Drosophila*)^22–24^ in *P. americana*. Genes from expanded gene families in *P*. *americana* are highlighted in red. Since all genes absent in *P*. *americana* were also found to be absent in other insect species, they were defined as *Drosophila*-specific components and outlined by gray dashed lines. 1, fas-associated DD protein; 2, death-related Ced-3/Nedd2-like protein; 3, effete; 4, TAK1-binding protein 2; 5, TGF-β-activated kinase 1; 6, kenny; 7, immune response deficient 5; 8, spätzle-processing enzyme; 9, myeloid differentiation primary response 88; 10, tube; 11, hopscotch; 12, socs36E; AMP, antimicrobial peptide; DOME, domeless; Dnr1, defense repressor 1; GNBP, Gram-negative binding protein; Gprk2, G protein-coupled receptor kinase 2; Grass, Gram-positive-specific serine protease; Iap2, inhibitor of apoptosis 2; IMD, immune deficiency; ModSP, modular serine protease; PGN, peptidoglycan; PGRP, peptidoglycan recognition protein; Pirk, poor IMD response upon knock in; Spz, spätzle; Spirit, serine protease immune response integrator; Stat92E, signal transducer and activator of transcription protein at 92E. Detailed information is additionally shown in Supplementary Tables 11–15. **b** Functional verification of genes in the Toll pathway against a classic Gram-positive bacterium, *Staphylococcus aureus*. Four major genes in the Toll pathway were knocked down by RNAi, compared to injection of control dsRNA (CK, a 92 bp non-coding sequence from the pSTBlue-1 vector). Corresponding mortality is shown upon *S. aureus* infection. Injection of cockroach saline solution (CSS, as a control) or *S. aureus* was performed 24 h after dsRNA injection. Error bars indicate standard deviation of three replicates. Bars labeled with different lowercase letters indicate significant difference between the two samples, with *p* < 0.05, one-way analysis of variance (ANOVA). **c** Functional relationship between the Toll pathway and expression of AMPs. *Dorsal*, a key component in the Toll pathway whose depletion caused the greatest mortality (shown in **b**), was knocked down by RNAi. Correspondingly, the relative expression of 11 antimicrobial peptide genes were measured and shown in **c**. Error bars represent s.d. of three replicates. Two-tailed Student's t-test: *p < 0.05; **p < 0.01

Our analyses then focused on gene families likely associated with the unique biology of the American cockroach and its success in adapting to urban environments. The American cockroach is an omnivorous scavenger and has adapted to human lifestyles and food sources. Adaptation to host and environment is mainly mediated by chemical communication and subsequent abilities to tolerate chemical and biological factors, such as toxins or pathogens. We therefore began analyses of signaling pathways that are involved in chemoreception, detoxification, and immunity (Figs. 2 and 3), which include expanded gene families relative to other insect species by automated analyses (Supplementary Tables 7 and 10).

Chemoreception systems provide attractive models to understand how organisms adapt to environments, because they lie between external environmental signals and internal physiological responses^10^. Chemosensory stimuli are mainly recognized by members of three related insect-specific chemosensory receptor families: olfactory receptors (ORs), gustatory receptors (GRs), and ionotropic glutamate receptors (IRs), while the odorant-binding proteins (OBPs) bind and transfer odors to ORs^11–13^. We manually annotated these gene families, and compared them among cockroaches, termites, and *Drosophila* (Fig. 2a–c; Supplementary Figs. 3 and 4). A total of 154 ORs were found in the *P*. *americana* genome, while other blattodean species were found with only half as many ORs (Fig. 2a; Supplementary Fig. 3). These expanded ORs could help *P*. *americana* to more easily detect traces (odors) of foods, especially fermenting foods, which the American cockroach prefers. Furthermore, we found 522 GRs in *P*. *americana*, which represents the greatest expansion of GRs in the insect species reported to date. Interestingly, 329 of these GRs formed a specific clade in the phylogeny and were annotated as potential bitter receptors (Fig. 2a, b). Being able to identify bitter tastes is generally considered as a self-protection system to tolerate bitter and toxic foods^14^, and expansion of bitter receptors has been observed in insect herbivores with adaptation to a great number of plant secondary metabolites^15^. The massive expansion of bitter receptors in *P*. *americana* may not only explain how this omnivorous and opportunistic species is able to adapt to diverse diets in a range of environments, but also enhance the potential value of this cockroach in the context of feeding habitat evolution, i.e., from omnivores to herbivores, in Blattodea. The IR gene family also has experienced a substantial expansion in the *P*. *americana* genome, in which we found a total of 640 candidate IRs (Fig. 2c), much more than that in the termite genome (148 in *Z. nevadensis*; Fig. 2a). IRs mediate neuronal communication at synapses throughout vertebrate and invertebrate nervous systems^16^. It was reported that IRs, in *Drosophila*, are expressed in neurons associated with coeloconic sensilla on the antenna and mediate responses to volatile chemical cues and temperature^16–18^. We propose that IRs might also greatly contribute to environmental adaptation in cockroaches. By contrast, we found the number of OBPs in blattodean species (with the most in *P. americana*) is dramatically reduced compared to *Drosophila* and other insects (Fig. 2a; Supplementary Fig. 4), suggesting the transport of odorant molecules may be functionally conserved in cockroaches. Considering these results, we hypothesize that substantial expansions in chemoreception families may contribute to the ability of the American cockroach to precisely discriminate environmental signals.

A detoxification system includes various enzymes and xenobiotic transporters that are crucial for insects to overcome numerous toxins^19^. We identified 178 cytochrome P450s, 90 carboxyl/choline esterases, 39 glutathione transferases, and 115 ATP-binding cassette transporters in *P. americana* (Fig. 2a, d; Supplementary Figs. 5–7). These associated families also show a pattern of general expansion in *P. americana*. We focused on P450s since their expansion is greatest in the American cockroach, compared with other blattodean species. Phylogenetic analysis of P450s across blattodean species clearly represents four major clans, i.e., the CYP2, the CYP3, the CYP4, and the mitochondrial (Mito) clade (Fig. 2d). We found that most P450 genes in *P. americana* are clustered with the lineages of CYP3 (79/178 genes) and CYP4 (62/178 genes) (Fig. 2d). The members of the CYP3 clan are highly diverse in their ability to metabolize a variety of naturally occurring compounds, and the expression of several genes in the CYP3 and CYP4 clans can be induced by various xenobiotics^20^. Thus, the expansions of these two clans may benefit cockroaches in insecticide resistance and survival in extreme conditions^21^.

Cockroaches generally live in moist and unsanitary areas and are particularly fond of fermenting foods^1^; thus, they have numerous opportunities to be exposed to microbes and pathogens. Insects exclusively rely on the innate immune system to combat infecting microbes. The humoral response of innate immunity is mediated mainly by three major signaling pathways: Imd^22^, Toll^23^, and Janus kinase-signal transducer and activator of transcription (JAK-STAT)^24^. Upon infection by Gram-negative bacteria as well as Gram-positive bacteria and fungi, the Imd and Toll pathways are activated, respectively, resulting in synthesis and secretion of antimicrobial peptides (AMPs) into the hemolymph, where they can kill invading microorganisms^25,26^. We found that all key components in the Imd, Toll, and JAK-STAT pathways, as well as effectors, are well represented in the *P. americana* genome. Compared with other insects, many genes in innate immunity, particularly in the Toll pathway, have been extensively expanded (Fig. 3a; Supplementary Fig. 8; Supplementary Tables 11–15). Gram-negative binding proteins (GNBPs) are pattern recognition proteins responsible for the detection of pathogens and the activation of the Toll pathway. We found 12 GNBP1-like and 2 GNBP3-like genes, more than in any of those insect species examined (maximum of 6 GNBPs in *Z. nevadensis*). The *Drosophila* genome encodes 9 Toll proteins, while *P. americana* genome encodes 14. Other components of the Toll pathway have also experienced gene duplications in *P. americana* (Fig. 3a and Supplementary Table 11, such as easter, spaetzle, pellino, pelle, and cactus). We identified 11 AMPs in *P. americana* genome, including defensins, termicins, attacin, drosomycin, Pro-rich peptide (Paprp-1), and Anti-fungus peptide (AFP) (Fig. 3a; Supplementary Table 15). We performed experiments by injecting the American cockroach with microbes to test the induction of AMPs by measuring antimicrobial activity of the cockroach crude extracts. We found strong antimicrobial activity after injection with *Escherichia coli* (Gram-negative bacterium), moderate antimicrobial activity with *Staphylococcus aureus* (Gram-positive bacterium), and weak antimicrobial activity with *Candida albicans* (fungi). Together, these findings suggest cockroach AMPs are potentially broad spectrum (Supplementary Fig. 9). Furthermore, injection of these microbes was able to upregulate the expression of all 11 AMPs to different degrees (Supplementary Fig. 10). Particularly high induction was observed for attacin and Paprp-1 after the injection with *E. coli*, defensin-3 and defensin-5 with *S. aureus*, and AFP and termicin-2 with *C. albicans* (Supplementary Fig. 10). We further investigated how the expanded Toll signaling pathway induces AMP expression and protects the American cockroach from microbial invasion. RNA interference (RNAi) knockdown of four important genes (*Toll-1A*, *Toll-1B*, *Myd88*, and *dorsal*) in the Toll pathway significantly increased the mortality of this cockroach upon *S. aureus* injection, with nearly complete mortality found after *dorsal* RNAi (Fig. 3b). We also examined the effect of *dorsal* RNAi on the expression of AMP genes. Importantly, *dorsal* RNAi not only significantly decreased the expression of defensin-3 and defensin-5, but also decreased the expression of most of the other AMP genes (Fig. 3c), which is consistent with the crucial role of the Toll pathway in innate immunity of *P. americana*. Taken together, our results provide solid evidence that the Toll pathway and AMPs play essential roles in the American cockroach in fighting invading pathogens. Importantly, dorsal and other key components in the Toll pathway could be potential molecular targets for controlling this serious pest of public health.

### Development and regeneration

High developmental plasticity is presumed crucial to the success of cockroaches to survive and succeed in many environments. Of all common cockroach species, the American cockroach has the largest body size, up to 53 mm in length; molts 6–14 times before metamorphosis; and has the longest lifecycle, up to approximately 700 days (Supplementary Fig. 1). Insect molting and metamorphosis are coordinately regulated by 20-hydroxyecdysone (20E) and juvenile hormone (JH), and JH prevents 20E-induced metamorphosis during the larval and nymph stages^27–30^. Insulin/insulin-like growth factors and 20E are the two major mechanisms that antagonize each other to define the final body size by regulating larval and nymph growth^31,32^. We found that crucial biosynthesis and signaling pathways for regulating insect development, such as 20E, JH, insulin, chitin metabolism, AMPK, and TOR (Supplementary Tables 16–21), are well represented in *P. americana*. Notably, we found two key genes involved in JH biosynthesis and metabolism (*Jhamt* and *Jhe*) and insulin-like peptide (*Ilp*) genes are substantially expanded (Supplementary Fig. 11). In addition, the cuticle protein family appears to be one of the most expanded gene families in *P. americana* (Supplementary Tables 7, 10 and 22).

**Fig. 4:**
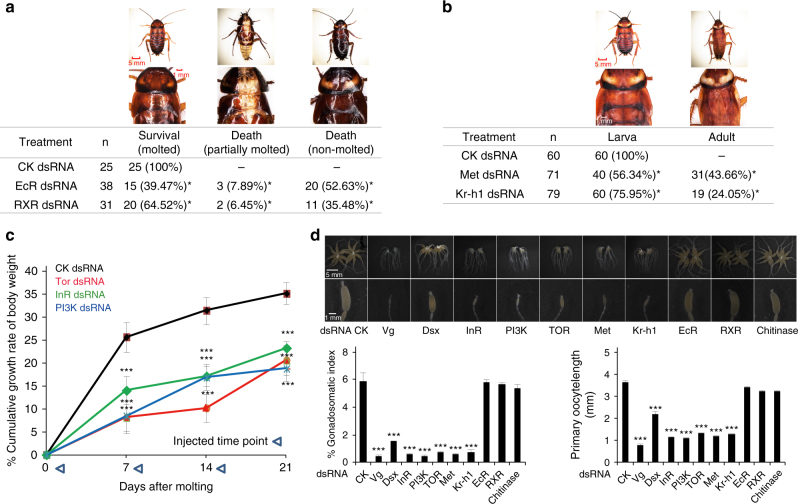
Functional studies of pathways regulating development and reproduction in the American cockroach. **a** Regulation of molting by ecdysone receptor (*EcR*) and retinoid X receptor (*RXR*) genes in the 20E signal pathway. Mortality rates were checked after RNAi treatment of indicated numbers of individuals (*n*). CK, control dsRNA corresponds to that in Fig. 3. **b** Regulation of metamorphosis by methoprene-tolerant (*Met*) and kruppel homolog 1 (*Kr-h1*) in the juvenile hormone (JH) pathway. Adult proportion index was checked after the JH singling was disrupted. **c** Regulation of growth by the insulin signaling pathway genes. Insulin-like receptor (*InR*), phosphoinositide 3-kinase (*PI3K*), and target of rapamycin (*TOR*) genes were repeatedly depleted by dsRNA injection during 3 weeks. Three injections were given and cumulative growth rate of body weight (%) was calculated. Student’s *t*-test: ***p < 0.001. **d** Morphology change of ovary maturation during the first reproductive cycle in virgin females. *Vitellogenin* (*Vg*), *double-sex* (*Dsx*), and nine genes involved in the pathways of insulin, JH, and 20E were knocked down by RNAi. Gonadosomatic index and primary oocyte length were used to evaluate the ovary maturation degree. All the data were calculated as the mean value of three replicates. Error bars represent standard deviation. Two-tailed Student’s *t*-test: *p < 0.05; ***p < 0.001. Details of all involved pathways are shown in Supplementary Tables 16–18

To understand how molting, metamorphosis, and growth are regulated by these upstream signals, we performed RNAi experiments to disrupt the 20E, JH, and insulin signals during the nymph stages of *P. americana*. We observed visible molting defects and eventually death upon RNAi knockdown of *EcR* and *RXR*, the two genes encoding the 20E nuclear receptor complex (Fig. 4a). We found precocious metamorphosis occurring when *Met* and *Kr-h1*, which encode the JH receptor and the JH downstream anti-metamorphic factor, respectively, were depleted by RNAi (Fig. 4b). The 11 *Jhamt* genes and 5 *Jhe* genes in *P. americana* genome should flexibly regulate JH titers and thus the number of molts (6–14), providing plasticity in metamorphic development. For the insulin signaling pathway, we examined three key genes (*InR*, *PI3K*, and *TOR*) and found that RNAi knockdown of each gene significantly retarded the growth rate of the nymphs (Fig. 4c). The expansion of the *Ilp* gene family (seven in the American cockroach) might mediate rapid growth of body size in the American cockroach when food resource is rich. These phenotypes suggest that 20E, JH, and insulin are mainly responsible for the regulation of molting, metamorphosis, and growth, respectively, although further investigation is required to understand their interactions.

The American cockroach reproduces periodically within the long adult stage, up to 600 days. In female adult insects, ovary maturation can be regulated by insulin, JH, or 20E by regulating vitellogenesis, DNA replication, cell proliferation, and generation of germline stem cells^33–35^. In some insect species, insulin signaling affects JH biosynthesis or JH signaling to regulate ovary maturation^36,37^. Note that facultative parthenogenesis is a reproductive strategy common in the American cockroach and termites, but absent in the German cockroach^38^, again supporting that the American cockroach is more closely related to the termites than to the German cockroach. A periodic reproductive cycle, including periodic ovary growth, oocyte maturation, and egg laying, was observed in newly emerged virgin females (Supplementary Fig. 12). We focused on how the gonadosomatic index and primary oocyte length in the first reproductive cycle are regulated by different upstream signals in virgin females. RNAi knockdown of *vitellogenin* (*Vg*) completely abolished ovary maturation, while RNAi knockdown of *double-sex* (*Dsx*, a transcriptional factor regulating *Vg* expression in many insects) had a less significant inhibition (Fig. 4d; Supplementary Fig. 12). Importantly, RNAi knockdown of key genes in the insulin pathway (*InR*, *PI3K*, and *TOR*) and the JH pathway (*Met* and *Kr-h1*) abolished ovary maturation (Fig. 4d), similar to the inhibitory effect of *Vg* RNAi (Fig. 4d; Supplementary Fig. 13), but RNAi knockdown of *EcR*, *RXR*, or *Chitinase* had no inhibitory effects (Fig. 4d). RNAi experiments in female adults demonstrated that both insulin and JH, but not 20E, are crucial for the regulation of ovary maturation, at least at the first reproductive cycle. Apparently, it is of interest to understand the molecular mechanisms underlying the interplay between insulin and JH during the first reproductive cycle as well as the hormone network connecting reproductive cycles. We suppose whether the American cockroach can grow fast or slow, molt more or less, and reproduce abundantly or not depends on its living conditions, which is consistent with its strong hormone-controlled adaptation ability.

**Fig. 5:**
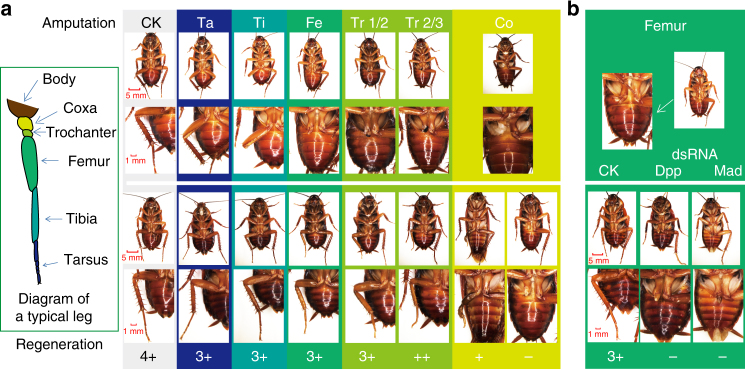
Leg regeneration ability and its regulation by the decapentaplegic (Dpp) pathway in *P. americana*. **a** Limb regeneration confirmation under different trauma severity indices. The diagram in the box represents a typical leg with indicated parts in which we performed the amputation experiment. Regeneration ability is shown in the bottom, according to the wild-type size. 4+, wild-type size; from 3+ to +, incomplete sizes; −, null. **b** Function of Dpp signaling pathway in leg regeneration. No leg regenerated (−) when *Dpp* and *mothers-against-dpp* (*Mad*) genes were knocked down by RNAi. CK, control dsRNA corresponds to that in Fig. 3

The American cockroach has a strong capability of limb regeneration during the nymph stages^39^, which is the main reason to call it “Xiao Qiang” in China. We re-examined its regeneration of missing leg segments after one molting by systematic amputation of the metathoracic limb, including all five podites (Fig. 5a). The ability of *P. americana* to regenerate the missing limb and the degree of recovery depend on the trauma severity indices (Fig. 5a). Importantly, trochanter and coxa are the two most important podites in leg regeneration, providing the possibility of studying the morphological and molecular details of cell proliferation and differentiation in this biological process (Fig. 5a). A number of important signaling pathways have been suggested to be involved in wound healing and tissue repair in *Drosophila* and vertebrates, including Decapentaplegic (Dpp), Jun N-terminal kinase (JNK), Grainy head (GRH), Wingless (Wg), Notch, Hippo, and Hedgehog (Hh)^40–43^. Based on our manual annotation, we found most key components of these seven pathways exist in the *P. americana* genome (Supplementary Tables 23–29), indicating that these pathways are evolutionarily conserved in insects. We also found gene expansions in the GRH, Wg, and Notch pathways (Supplementary Fig. 10; Supplementary Tables 25–27) in *P. americana* and speculated that these pathways may contribute to the impressive ability of this cockroach to regenerate lost appendages. Dpp and Mad (mothers-against-dpp) are the ligand and downstream transcription factor, respectively, in the Dpp pathway. We next used RNAi to reduce the expression of *Dpp* and *Mad* to determine their roles in leg regeneration. When the tarsus–tibia–femur segments were removed and these two genes were depleted by RNAi, the regeneration of missing limbs was completely prohibited after one molting (Fig. 5b). These results show that the Dpp pathway is necessary for wound healing and tissue repair during cockroach leg regeneration, which is considerably helpful for cockroaches to recover from the body damage or hurt. As mentioned above, *Kang Fu Xin Ye*, an ethanol extract of the American cockroach, has been developed as a prescribed drug for wound healing and tissue repair. We are currently investigating whether there is a “growth factor” (Supplementary Table 30) connecting leg regeneration in the American cockroach to its ethanol extract that is used for wound healing and tissue repair in humans.

In summary, our genomic and functional analyses in the American cockroach provided insights into its success in the adaptation to urban environments and the biology of developmental plasticity in cockroaches. High efficiency of RNAi in the American cockroach unlocks its potential as a genetic model system for investigating cockroach biology. The harm of American cockroaches is becoming more serious with the threat of global warming. Our study may shed light on both controlling and making use of this insect. A paucity of genomic resources in Blattodea has precluded the discovery of evolutionary signatures of euociality in this hemimetabolous clade. The American cockroach system should be informative for addressing eusociality and feeding habitats in Blattodea, given that it has a closer relationship with termites than the German cockroach. Genomic studies of additional species in Blattodea should be forthcoming, especially the subsocial and wood-feeding species in *Cryptocercus* (Cryptocercidae), to illuminate the transition from cockroaches to termites.

## Methods

### Animals

The line of *P. americana* (the American cockroach) was provided by Dr. Huiling Hao, whose line has been maintained with inbreeding for 30 years. Samples of *P. australasiae* (the Australian cockroach) and *P. fuliginosa* (the smokybrown cockroach) were provided by Professor Jianchu Mo (Zhejiang University, China). The colony was kept at 29 °C and a relative humidity of 70–80% in plastic cages and animals were fed commercial rat food and water at libitum. To obtain pools of synchronized animals, newly molted male and female adults and mid-age larvae were picked from the colony, respectively, and placed in separate containers and provided with rat food and water. Operations were performed after anaesthetizing with CO_2_. For treatment, three biological replicates were performed with 30 animals involved in each replicate.

### Genome and transcriptome sequencing

DNA for de novo sequencing was all extracted from a single female adult. Libraries of stepwise increased inserts were constructed following the standard protocol. All libraries were sequenced on Illumina sequencing platforms by Berry Genomics Co. Ltd. In summary, a total of 1.06 Tb data were generated for three species of *Periplaneta* (Supplementary Table 1). To examine the transcriptome, we also sequenced full-length transcripts using the PacBio sequencing system (Pacific Biosciences). A total of 17.5 Gb transcriptome sequencing data were generated from three independent libraries, with inserts of 1–2 Kb, 2–3 Kb, and 3–6 Kb, for the messenger RNA pool of all stages of cockroach development (Supplementary Table 1).

### Genome assembly

Initial assemblies were generated by DiscovarDeNovo (v52488; https://software.broadinstitute.org/software/discovar/blog/) with default settings. SSPACE (v3)^44^ was then used to join contigs into scaffolds based on linking evidence of mate pair libraries, step by step ranging from 600 bp to 13 kb insert size, under default parameters. Gaps within these scaffolds were iteratively filled with paired-end reads of short-size inserts using GapCloser available in SOAPdenovo^45^. Main properties of the resulting assembly are comparable to other blattodean species and related species of large genome size. Notably, our assembly presents the longest N_50_ size in contigs compared with other related species (Supplementary Table 2). We evaluated the completeness of our assembly using sequence of different independent resources, which include both nucleotide sequences of *P. americana* itself and protein sequence of proposed conservation across species, as follows: long transcriptomic reads, with a mean length of 1.5 Kb, that were generated by PacBio; de novo assembled transcripts, based on Illumina sequencing reads (SRA#DRP001319) using Trinity r20140717 with default settings; expressed sequence tags downloaded from NCBI (National Center for Biotechnology Information); uploaded nucleotide sequences of *P. americana* in GenBank; 458 CEGMA^46^ proteins (copies of *Drosophila*) that were recognized as potential conserved core proteins across species. These sequences were aligned back to the assembled genome using genblastA v1.0.1 with “-e 1e-10 −r 1”^47^ and their recovered proportions were subsequently calculated by an in-house PERL script. BUSCO v3^48^ was also utilized in quantitative measuring for the assessment of genome assemblies, using insecta_odb9 as lineage input. The evaluation properties of the American cockroach genome assembly were presented in Supplementary Tables 2–4.

### Genome annotation

Repeats, including repetitive sequences and transposable elements, were identified using RepeatMasker v 4.0.5^49^ (http://www.repeatmasker.org) against both de novo repeat library, which was built by RepeatModeler v1.0.4 (http://www.repeatmasker.org), and the arthropod set of Repbase v20140131^50^. The official gene set (OGS1; Supplementary Table 4) was based on a GLEAN consensus model^51^, which combined transcriptome (*Periplaneta*), homology (*Blattella*^5^, *Zootermopsis*^6^, *Locusta*^4^, *Drosophila*, and UniProt), and ab initio sets (AUGUSTUS^52^, SNAP^53^, and GENSCAN^54^) as described^55^. Model training for each ab initio program was initially based on assembled transcripts and followed with an iteratively trained approach. Automatic annotations were performed using BLASTP (E < 10^−5^) against a number of public databases as listed in Supplementary Table 5. Local run of InterProScan v5.13-52.0^56^ with all implemented methods was utilized to assign domains, Gene Ontology terms, as well as KO terms to gene models.

### Orthology and phylogenomics

A total of 12 representative insect species, including *P. americana*, were selected for orthology analysis. Related protein sets of involved species are listed in Supplementary Table 6. Automatic orthology was determined using the OrthoMCL pipeline^57^. In brief, we first filtered out redundant splice variants to keep the longest isoform for each protein set; then, all-against-all protein comparisons were performed using BLASTP with E < 10^−5^; finally, High-scoring Segment Pair (HSPs) were processed by MCL v10-201 to define orthologs, inparalogs, and co-orthologs. A total of 538 exactly single-copy universal genes were used to reconstruct the phylogeny. Multiple alignments of protein sequences for each ortholog group were performed using Muscle v 3.8.31^58^. Of these alignments, the conserved blocks were extracted using Gblocks v 0.91b^59^ and subsequently concatenated to 12 super genes with 75,839 amino acids. The species tree was calculated using PhyML v3.1^60^ with the WAG model with 100 replicates of bootstrap analysis.

### Genome-wide sequence identity

We focused on pairwise sequence identity in amino acids between species of Blattodea, including *B. germanica*^5^ (v0.5.3), *Z. nevadensis*^6^ (v2.2), and three species of *Periplaneta* in this study. For *P. australasiae* and *P. fuliginosa*, we first de novo assembled preliminary contigs using idba v1.1.1^61^ following default parameters. All protein-coding genes were then aligned to each set of contigs using genblastA v1.0.1 with “-e 1e-5 -r 1”^47^. GeneWise v2.2.0^62^ was finally utilized to predict the gene models in these two genomes, respectively, for each input seed of *P. americana*. Orthology across these blattodean species was analyzed as described above. In order to exclude potential biases, we limited the comparison of sequence identity within the commonly aligned blocks across all analyzed species, which were generated by MUSCLE^58^ and Gblocks^59^ as described above. Sequence identity was directly calculated by an in-house PERL script. Nonparametric regression was calculated using the lowess function implemented in R.

### Gene families

We performed manual curation for each gene of the families and pathways that we addressed in this study; approximately 2000 genes of the special interest in the biology of the American cockroach were manually annotated. Most gene families were based on known models in *Drosophila*. For informative comparison, we included the corresponding information of the *B. germanica* gene set^5^. To avoid discordant information between these two studies^5^, we used the currently latest version of the gene set and associated annotation, i.e., v0.6.2, of *B. germanica*. Multiple alignments were performed by ClustalX v2.2.0^63^ and manually curated for well-aligned blocks. Phylogenetic analysis was conducted using the maximum-likelihood algorithm and JTT model implemented in MEGA7^64^ for 100 replicates of bootstraps.

Because identification of chemosensory receptor genes is commonly problematic by automated predictions, we identified this class of genes in the genomes using TBLASTN searches with known receptors of *Drosophila* and *Zootermopsis* as queries, followed by iteration. Gene structures within the genomic loci with significant hits (E < 10^−5^) were predicted using GeneWise v2.2.0^62^ with pseudogene masked. We carefully checked the multiple alignments and removed poor aligned regions before phylogeny analysis. Given the large data set of involved sequence, we used neighbor-joining algorithm implemented in MEGA7^64^ for 100 replicates.

For comparisons in large gene families, different approaches and/or cutoffs in independent studies may cause systematic bias. Thus, we utilized a consensus approach to re-identify large gene families in published genomes to generate a comparable result in this study, such as chemoreceptors and detoxification-related gene families. We note that these annotations do not represent the official genome annotations of the corresponding species. Gene function enrichment analysis was conducted using an online resource (http://www.omicshare.com/), under the default instructions.

### AMP induction

Bacteria of *E. coli* (PTA-5952), *S. aureus* (ATCC29213), and *C. albicans* (ATCC29213) were cultured in LB liquid medium. Overnight bacteria cultures were centrifuged and resuspended in the cockroach saline solution (CSS)^65^ containing *E. coli* (OD_600_ = 0.25), *S. aureus* (OD_600_ = 1.0), and *C. albicans* (OD_600_ = 1.0). For AMP induction, adult male American cockroaches emerged within 10 days were injected using sharp micro syringe in posterolateral abdomens with 3 bacterial suspensions of *E. coli*, *S. aureus*, *C. albicans*, or CSS.

### Crude purification

For crude purification of AMPs, the whole animals were ground to a fine powder in the constant presence of liquid nitrogen to prevent protease degradation. The powder was then transferred in 10 volumes (mass/vol.) 0.1 M acetic acid and extracted for 24 h under gentle shaking^66^. After centrifugation at 13,000 ×* g* for 15 min again, the supernatant was boiled for 15 min, and then centrifuged at 13,000 × *g* for 15 min again, the supernatant was collected followed by drying in vacuum drying oven, then resuspended in 0.1 vol. (the volume of supernatant without drying treatment) double distilled water to obtain AMP crude extracts.

### Assay of antimicrobial activity

The radial diffusion assay was performed to test the antimicrobial activities of AMP crude extracts, as described previously with slight modifications^66^. Sterile Petri dishes (9 cm diameter) received 7.5 ml of LB solid medium, containing ~2 × 10^5^ logarithmic-phase cells of a given bacterial strain. Wells (5 mm diameter) were cut into the freshly poured plates after the solidification of the agar. Each well received a 20 μl aliquot of the fraction expected to contain antibacterial molecules. The plates were incubated overnight at 37 ℃, and the diameters of the clear zones were recorded, after subtraction of the well diameter.

### RNAi in vivo

For RNAi, 300–500 bp fragments of the target genes were PCR amplified from the complementary DNA. Then, the T7 promoter sequence attached primers were used for PCR amplification of the double-stranded RNA (dsRNA) templates. The dsRNA was synthesized using T7 RiboMAX Express RNAi kit (Promega) according to the manufacturer’s instructions^67^. A 92 bp non-coding sequence from the pSTBlue-1 vector (dsCK) was used as control dsRNA^68^. dsRNA was quantified using a Nanodrop spectrophotometer (Thermo Scientific) and was injected into the abdomen of cockroaches. All dsRNA synthesis primers used in this study are listed in Supplementary Table 31. In the experiments on innate immunity, for each gene, newly emerged adult males within 10 days were injected with a dose of 3 μg dsRNA per animal. In the experiments on molting, metamorphosis, and growth, newly emerged mid-age larvae were injected with a dose of 3 μg dsRNA per animal at 0, 7, and 14 days after molting. In the experiments on ovary development, newly emerged adult females were injected with 3 μg dsRNA per animal at 2, 4, and 6 days after eclosion. In the experiments on limb regeneration, the section below the femur was amputated, and dsRNAs were injected one day before amputation, then injection was operated once every other day for four times. Controls were injected with the same volume and dose of dsCK. Three biological replicates were performed. The efficiencies of RNAi are listed in Supplementary Fig. 2.

### Total RNA extraction and quantitative real-time PCR

After 24 h upon dsRNA injection or microorganism infection, abdominal fat body tissues of cockroaches were collected, and total RNA was extracted using EASYspin Plus RNA extraction kit (Aidlab, China) according to the manufacturer’s instructions. Reverse transcription was performed by using Reverse Transcriptase M-MLV (Takara) as previously described^69^. Quantitative real-time PCR (qRT-PCR) was performed using SYBR^®^ Select Master Mix (Applied Biosystems) and the Applied Biosystems™ QuantStudio™ 6 Flex Real-Time PCR System. *Actin* was chosen as a reference gene for qRT-PCR analysis^68^. All qRT-PCR primers used in this study are listed in Supplementary Table 31.

### Amputation and microscopy observation

The tarsus, tibia, femur, trochanter, coax of 20 mid-age larvae were amputated in 2 days after molting, respectively. Sections and regeneration legs were photographed using a Nikon DS-Ri2 camera. Cockroaches were dissected in CSS, under an OLYMPUS SZ61 microscope. For each female animal, the ovary was dissected on day 7; body weight, ovarian weight, and primary oocyte lengths were recorded at the same time, respectively. Images of ovary and ovariole were captured with a Nikon DS-Ri2 camera and Nikon SMZ25 microscope. The length of ovarioles was measured using NIS-Elements BR 4.50.00 software. For larvae, fat body and coxa were collected at 48 h post injection of dsRNA and flash-frozen in liquid nitrogen to prevent RNA degradation and stored at −80 ℃ until further processing. For all experiments described in this paper, at least three biological repeats were performed, and 30 animals were used in each biological repeat.

### Data availability

All sequence data from the American cockroach genome project have been deposited at DDBJ/ENA/GenBank under the accession PGRX00000000. All relevant data are available from the authors.

## Electronic supplementary material

Supplementary Information
