# Supplementary material for: The genomic and functional landscapes of developmental plasticity in the American cockroach
Source: Nat Commun. 2018 Mar 20;9:1008. doi: 10.1038/s41467-018-03281-1 (PMC5861062; doi:10.1038/s41467-018-03281-1)
Supplement: Supplementary file 1 — Supplementary Information [file 41467_2018_3281_MOESM1_ESM.pdf]

Supplementary Information

The genomic and functional landscapes of developmental plasticity in the American cockroach

Li et al.

Contents

Supplementary Fig. 1 4
Supplementary Fig. 2 5
Supplementary Fig. 3 6
Supplementary Fig. 4 7
Supplementary Fig. 5 8
Supplementary Fig. 6. 9
Supplementary Fig. 7 10
Supplementary Fig. 8 11
Supplementary Fig. 9 12
Supplementary Fig. 10 13
Supplementary Fig. 11 14
Supplementary Fig. 12 15
Supplementary Fig. 13 16
Supplementary Tables 17
Supplementary Table 1........ 17
Supplementary Table 2........ 18
Supplementary Table 3........ 19
Supplementary Table 4........ 20
Supplementary Table 5........ 21
Supplementary Table 6........ 22
Supplementary Table 7........ 23
Supplementary Table 8........ 24
Supplementary Table 9........ 25
Supplementary Table 10........ 26
Supplementary Table 11........ 27
Supplementary Table 12........ 30
Supplementary Table 13........ 33
Supplementary Table 14........ 34
Supplementary Table 15........ 35
Supplementary Table 16........ 36

Supplementary Table 17 38
Supplementary Table 18 42
Supplementary Table 19 50
Supplementary Table 20 53
Supplementary Table 21 57
Supplementary Table 22 61
Supplementary Table 23 62
Supplementary Table 24 64
Supplementary Table 25 65
Supplementary Table 26 66
Supplementary Table 27 68
Supplementary Table 28 70
Supplementary Table 29 72
Supplementary Table 30 73
Supplementary Table 31 75
Supplementary References 77

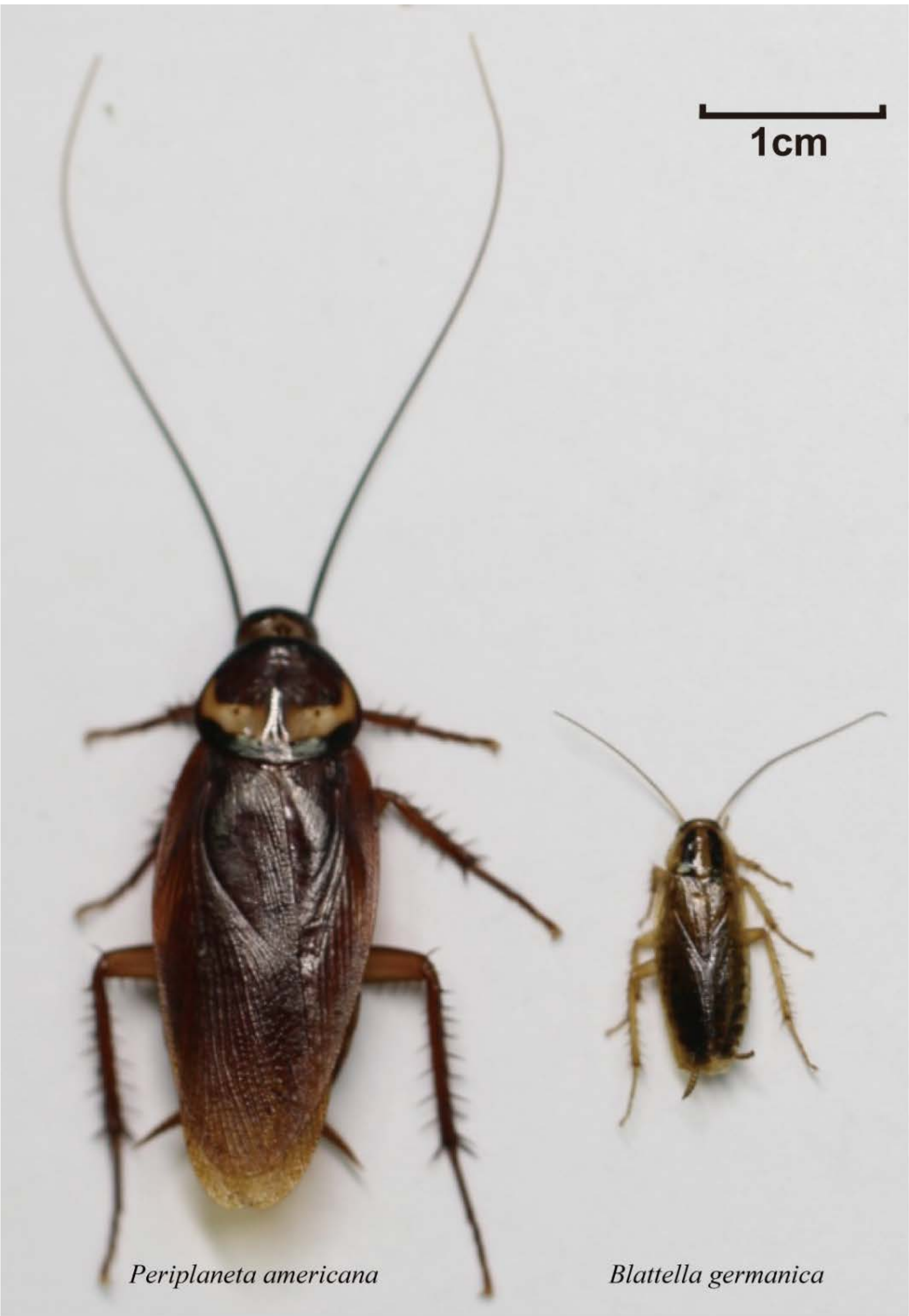

Supplementary Fig. 1. Images of female adults of the American cockroach, *Periplaneta americana*, and the German cockroach, *Blattella germanica*.

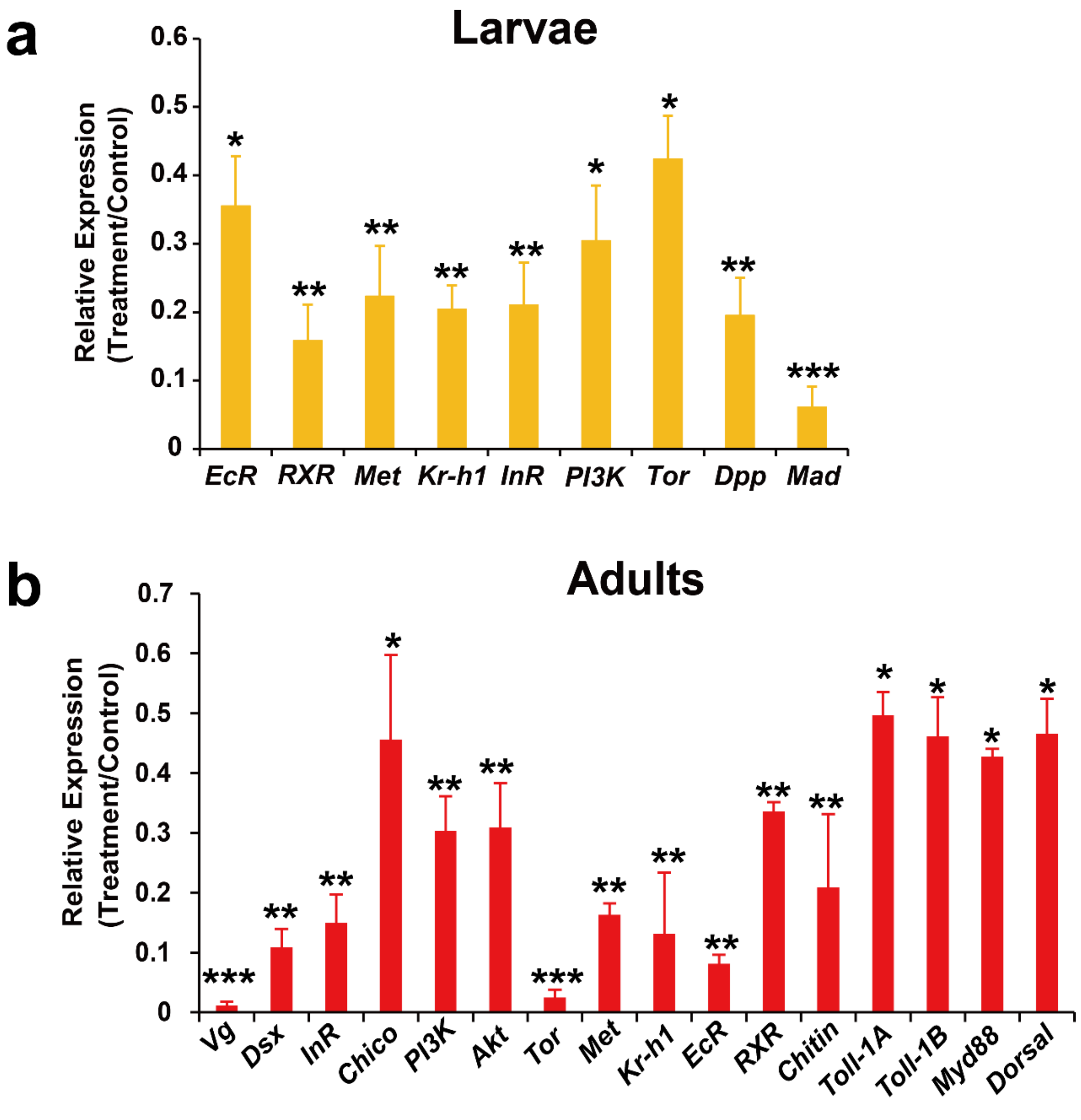

Supplementary Fig. 2. The mRNA levels of RNAi target genes were significantly decreased after corresponding dsRNA injection in both larvae (a) and adults (b). The data were calculated as the mean value of three duplications. Error bars represent standard deviation. Student's t-test: *$p < 0.05$; *$p < 0.01$; ***$p < 0.001$.

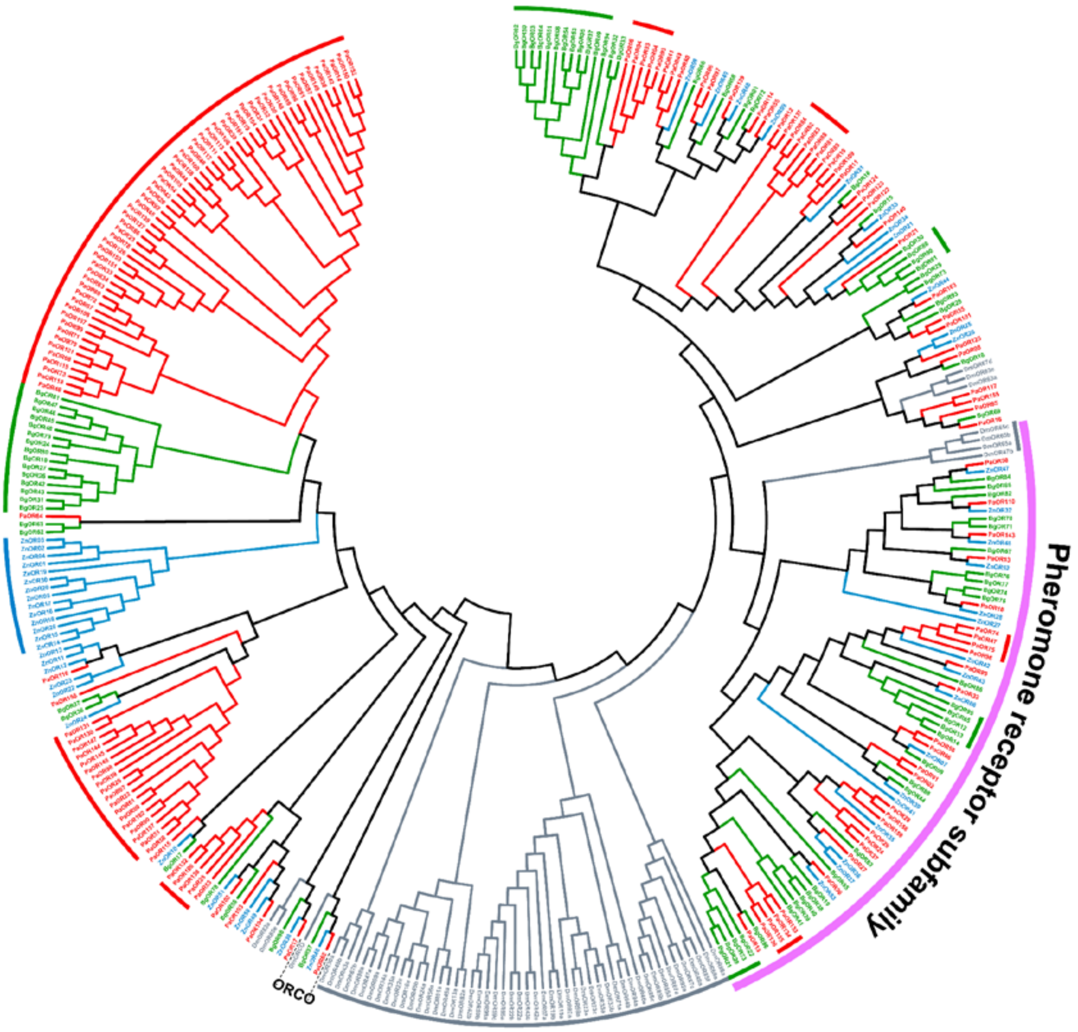

Supplementary Fig. 3. Phylogenetic tree of the olfactory receptors (ORs). The phylogenetic analysis was inferred using the Neighbor-joining method involving 353 amino acid sequences. All ambiguous positions were removed for each sequence pair. Color corresponds to that in **Fig. 2**.

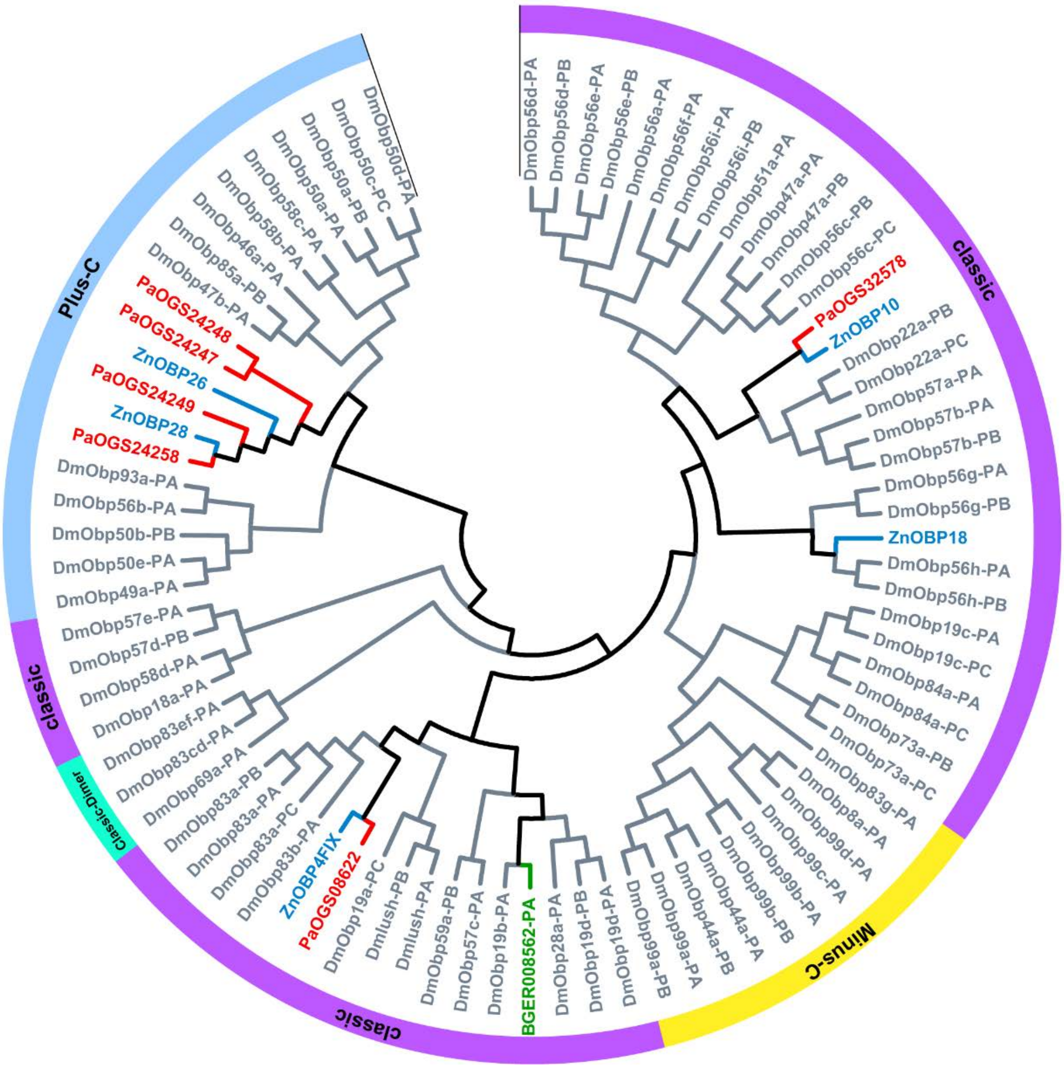

Supplementary Fig. 4. Phylogenetic tree of the odorant-binding proteins (OBPs). The Neighbor-joining analysis involved 84 amino acid sequences. Color corresponds to that in **Fig. 2**.

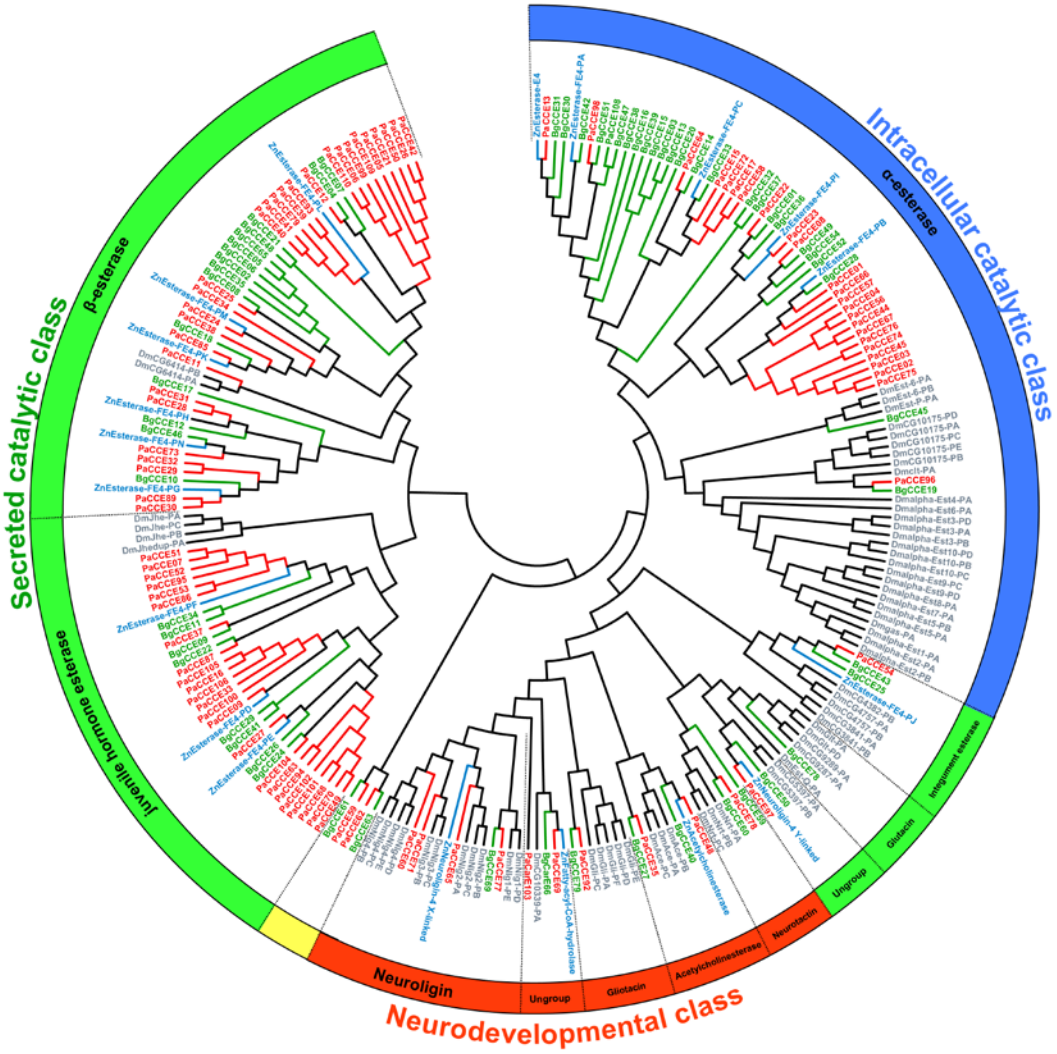

Supplementary Fig. 5. Phylogenetic tree of the carboxyl/cholinesterase (CCE) genes. The Neighbor-joining analysis involved 237 amino acid sequences. Color corresponds to that in **Fig. 2**.

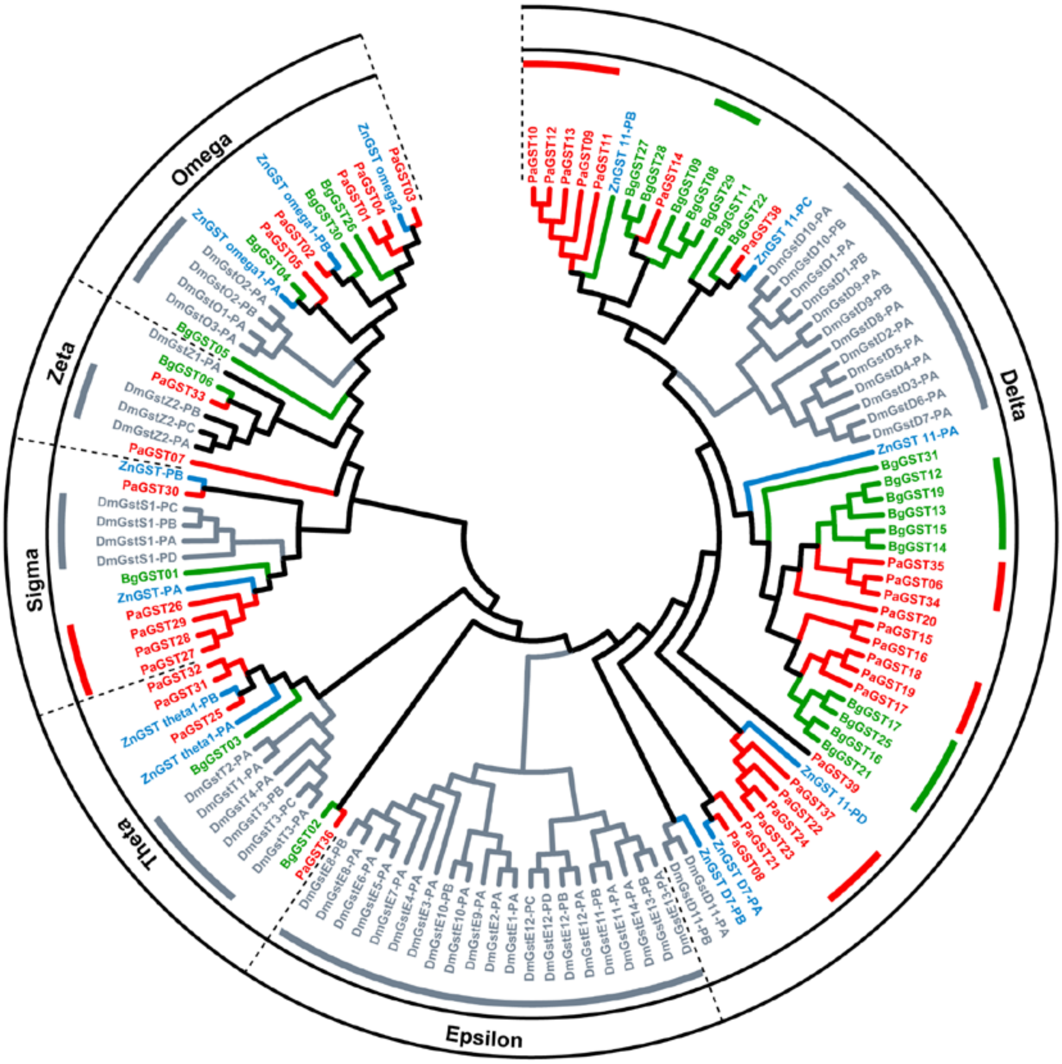

Supplementary Fig. 6. Phylogenetic tree of the glutathione-S-transferase's (GST) genes. The Neighbor-joining analysis involved 361 amino acid sequences. Color corresponds to that in **Fig. 2**.

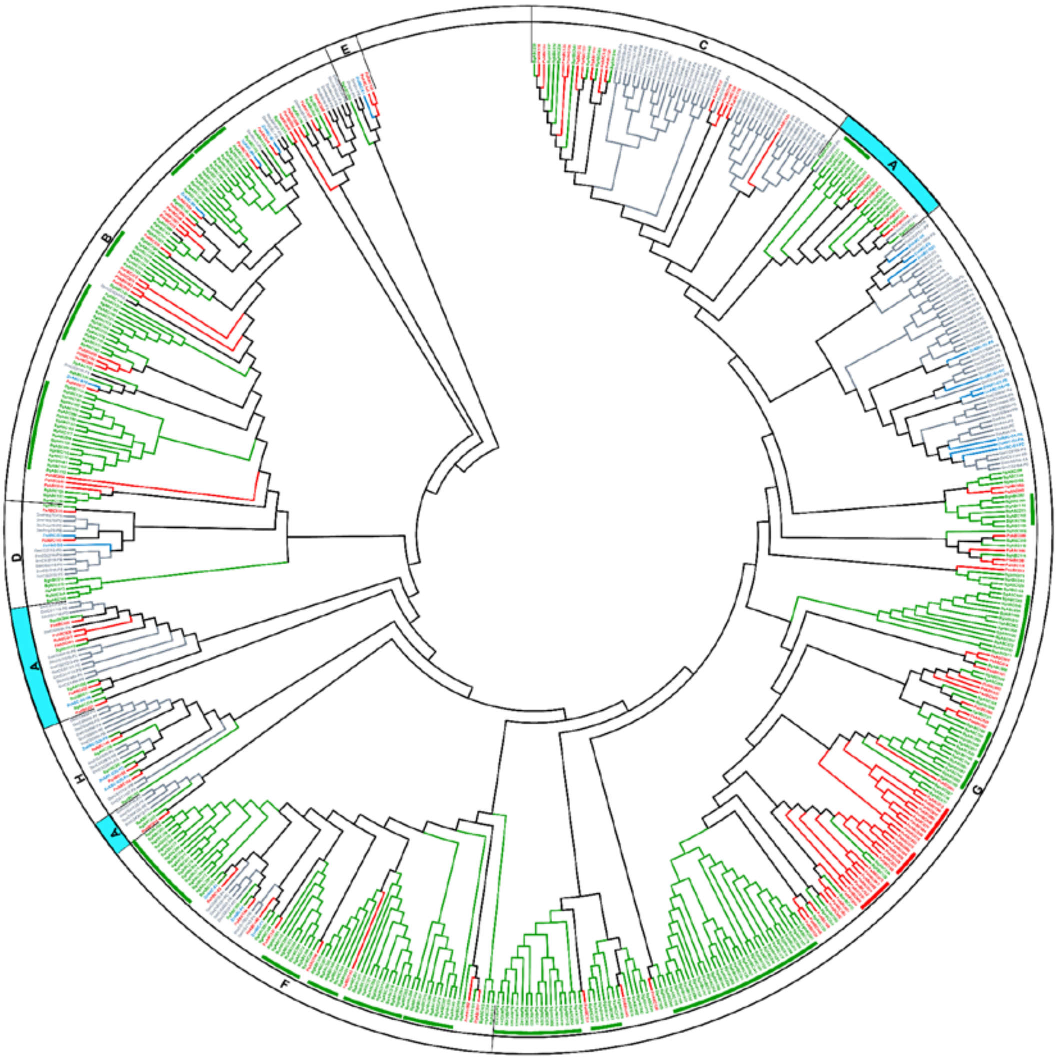

Supplementary Fig. 7. Phylogenetic tree of the ABC transporters. The Maximum Likelihood analysis involved 578 amino acid sequences. The bootstrap consensus tree inferred from 100 replicates is taken to represent the evolutionary history of the taxa analyzed. Branches corresponding to partitions reproduced in less than 50% bootstrap replicates are collapsed. Initial tree(s) for the heuristic search were obtained automatically by applying Neighbor-Join and BioNJ algorithms to a matrix of pairwise distances estimated using a JTT model, and then selecting the topology with superior log likelihood value. The analysis involved 578 amino acid sequences. Color corresponds to that in **Fig. 2**.

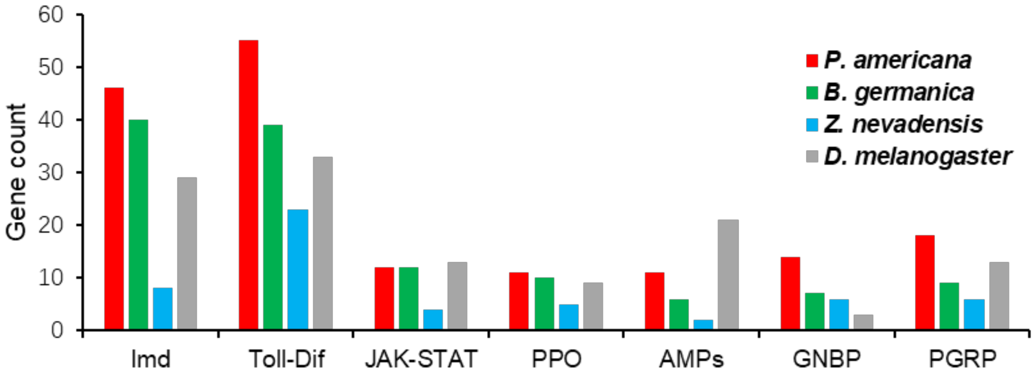

Supplementary Fig. 8. Counts of immune-related genes in the genomes of three Blattodea species and *Drosophila*. Imd, Immune Deficiency pathway; Toll, Toll-DIF pathway; JAK-STAT, Janus kinase/signal transducers and activators of transcription pathway; PPO, Insect prophenoloxidase pathway; AMPs, Antimicrobial peptides; GNBP, Gram-negative binding proteins; PGRP, peptidoglycan-recognition proteins.

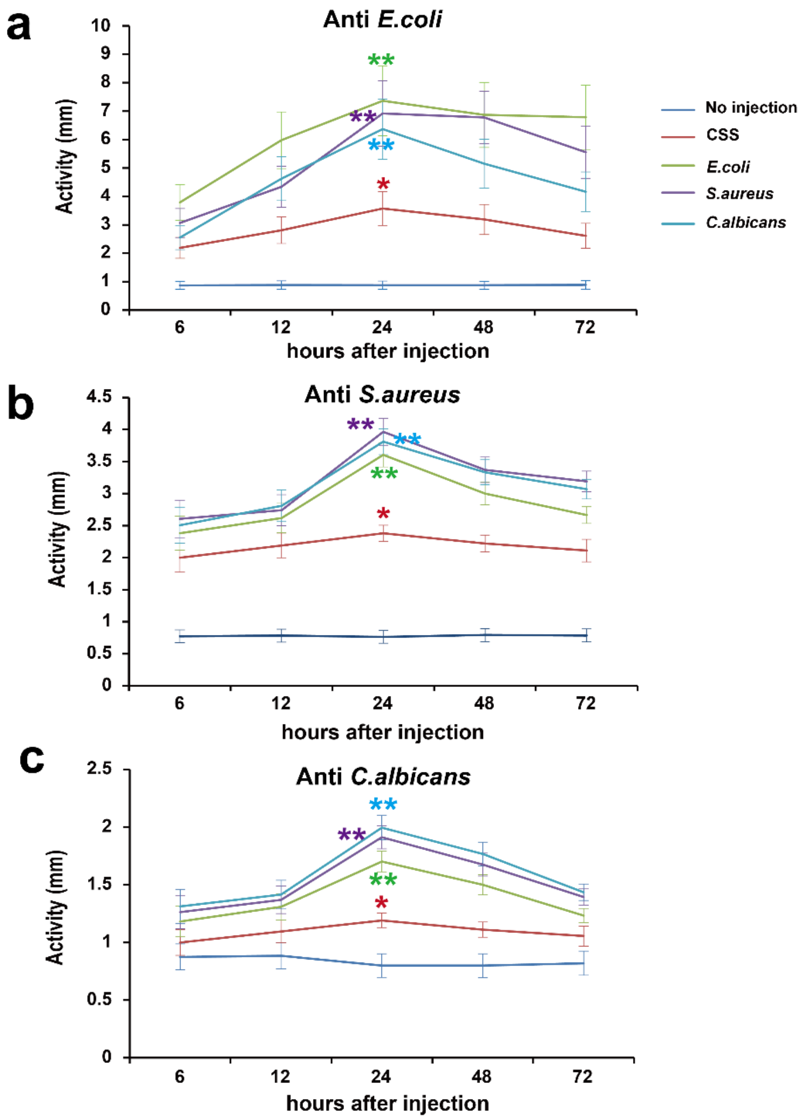

Supplementary Fig. 9. Test the induction of AMPs by measuring antimicrobial activity of the cockroach crude extracts after microbe injection. The changes of antimicrobial activity against *E. coli* (a), *S. aureus* (b), and *C. albicans* (c) in the cockroach crude extracts from 6 h to 72 h after injection of CSS, *E. coli*, *S. aureus*, or *C. albicans*. No injection is used for control. The peaks arrive 24 h after injection. Antimicrobial activity is expressed in diameter (mm) of growth inhibition zone. All the data were calculated as the mean value of three replicates. Error bars represent standard deviation. Student's t-test: *$p < 0.05$; *$p < 0.01$; ***$p < 0.001$.

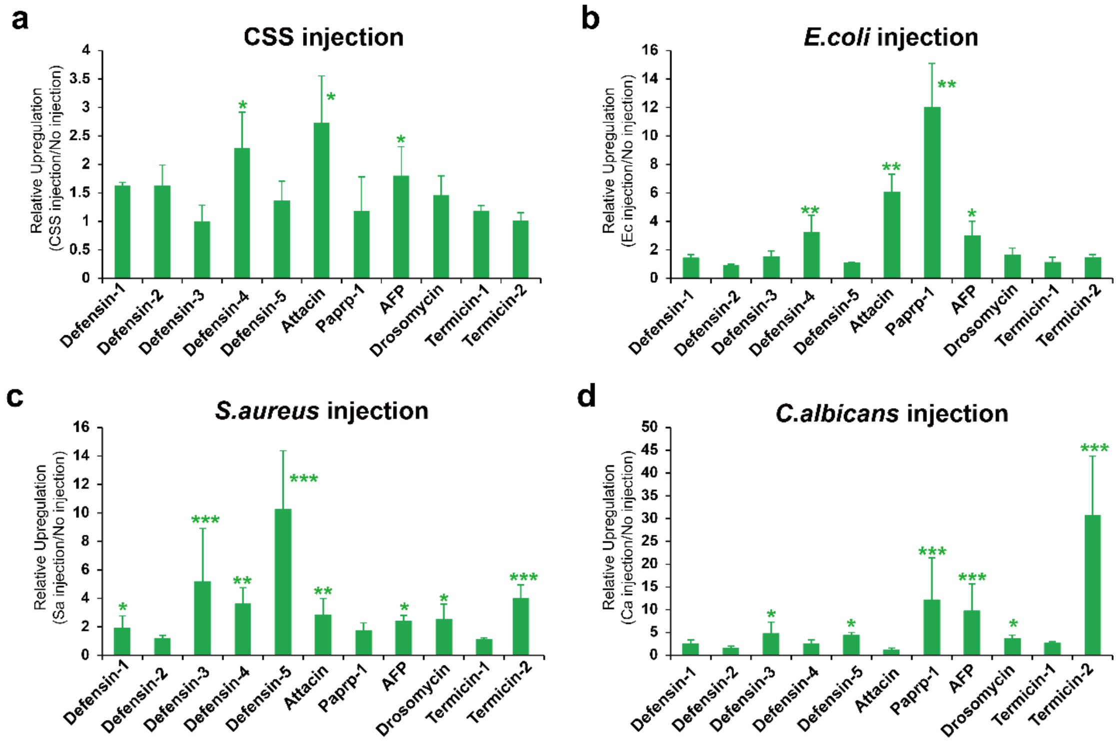

Supplementary Fig. 10. Compared to no injection animals, the mRNA levels of 11 AMPs genes are increased after 24 h of injection with CSS (a), *E.coli* (b), *S.aureus* (c), or *C.albicans* (d).All the data were calculated as the mean value of three duplications. Error bars represent standard deviation. Student's t-test: *$p < 0.05$; *$p < 0.01$; ***$p < 0.001$.

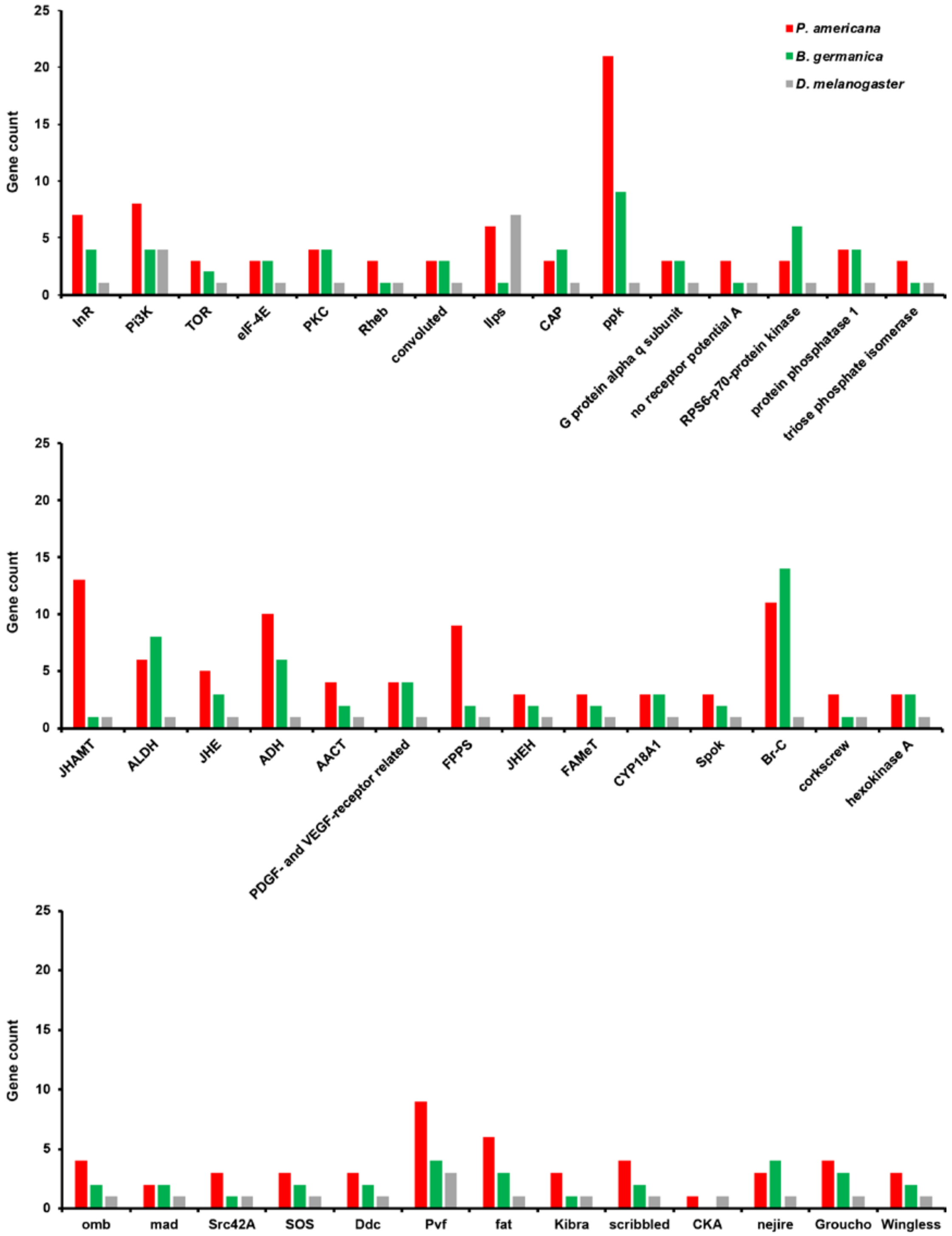

Supplementary Fig. 11. List of expanded gene families that are involved in development and regeneration in *P. americana*. These genes were annotated based on orthology.

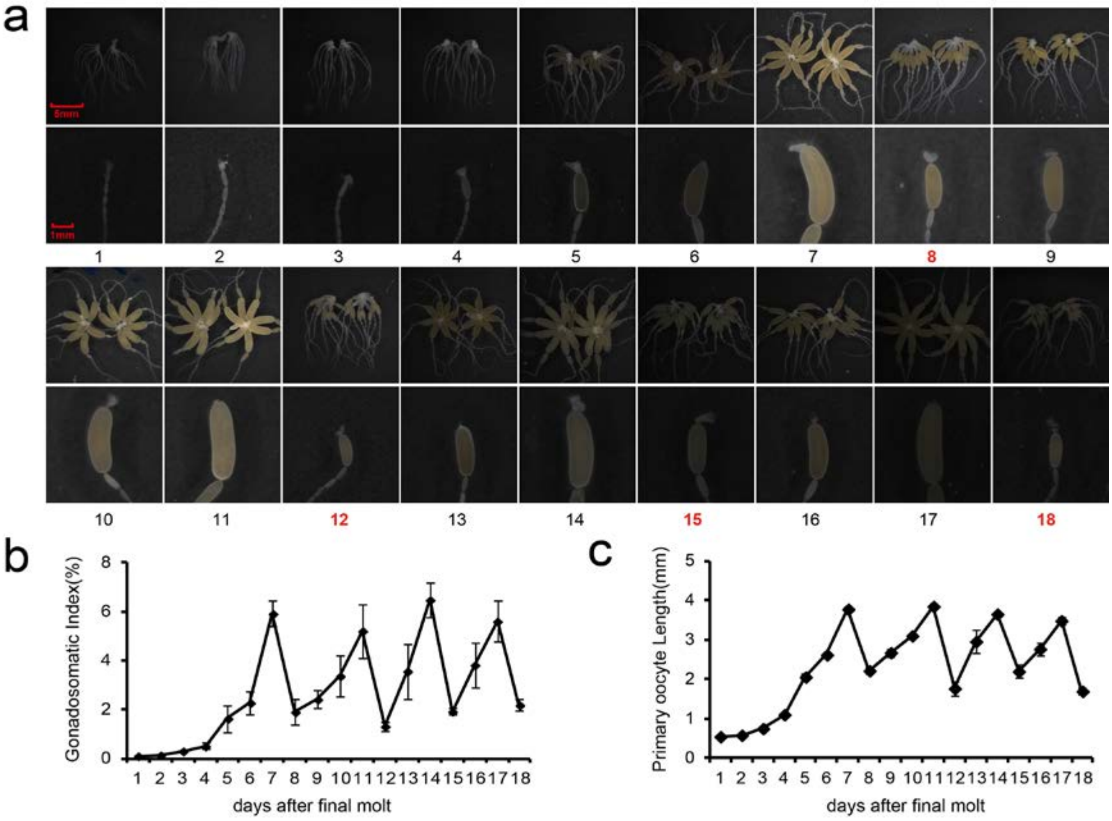

Supplementary Fig. 12. Periodically reproductive cycle of ovary in virgin females (a). Whole ovary gonadosomatic index (b) and primary oocyte length (c) were measured. The data were calculated as the mean value of three duplications. Error bars represent standard deviation.

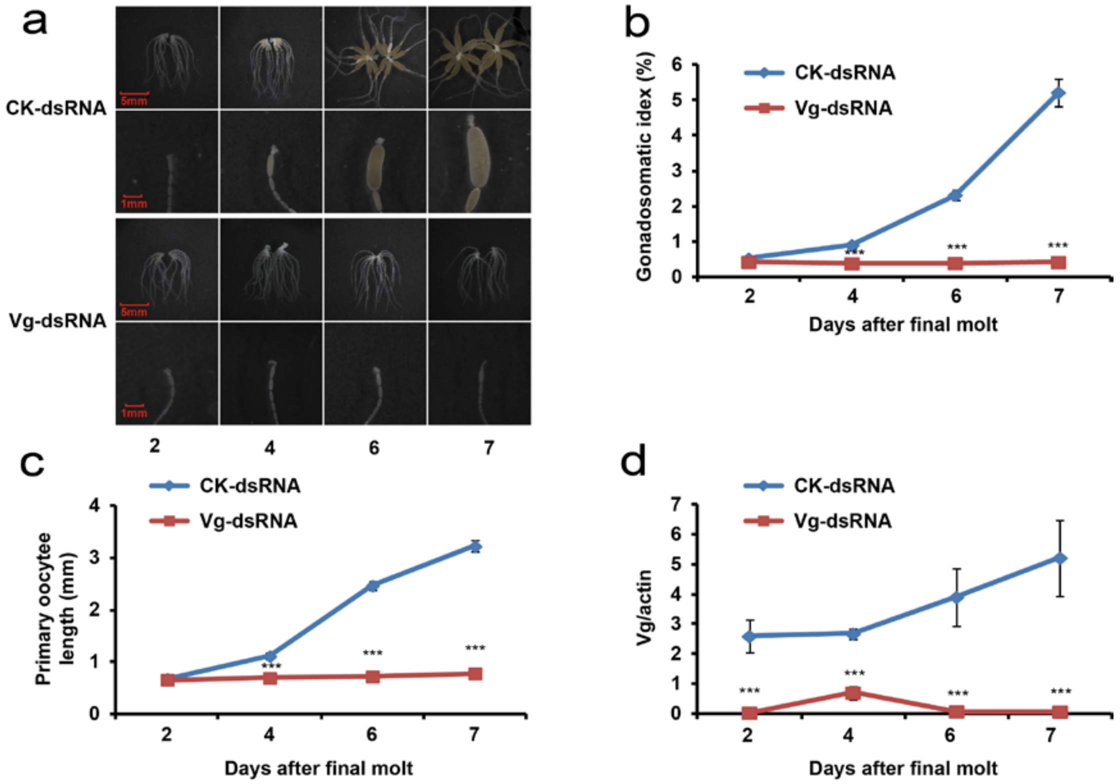

Supplementary Fig. 13. Ovary development process under normal and *Vg* disruption conditions in the first reproductive cycle. RNAi knockdown of *Vg* completely abolished ovary maturation (a). Whole ovary gonadosomatic index (b), primary oocyte length (c) and Vg/Actin index (d) were calculated in four-time points. The data were calculated as the mean value of three duplications. Error bars represent standard deviation. Student's t-test: *$p < 0.05$; *$p < 0.01$; ***$p < 0.001$.

Supplementary Tables

Supplementary Table 1 Statistics of sequencing data

Library size (bp)	Sequencing length (bp)	Sequencing data (Gb)	Estimated coverage (x)
P. americana			
250	2 * 150	102.2	36.5
450	2 * 250	274.7	98.1
600	2 * 150	157.7	56.3
3,000	2 * 150	86.3	30.9
5,000	2 * 150	88.6	31.6
8,000	2 * 150	56.8	20.3
13,000	2 * 150	59.7	21.3
	**Total**	**826.1**	**295.0**
P. australasiae			
250	2 * 150	125.6	44.9
P. fuliginosa			
250	2 * 150	113.1	40.4
Transcriptome of *P. americana*			
1-2 Kb	Full length	5.4	-
2-3 Kb	Full length	4.9	-
3-6 Kb	Full length	7.2	-

Supplementary Table 2 Common features of genome assemblies

	Pame[1]	Bger[1]	Znev[1]	Mnat[1]	Lmig[1]
Assembly size (Mb)	3,380	2,021	493	1,172	6,908
% Gap	4.4	16.3	4.3	4.9	15.8
Contig N50 (Kb)	21	12	20	16	9
Scaffold N50 (Kb)	333	1,066	740	1,997	326
Scaffold N60 (Kb)	260	834	585	1,578	190
Scaffold N70 (Kb)	198	608	431	1,198	111
Scaffold N80 (Kb)	143	394	303	829	59
Scaffold N90 (Kb)	92	154	117	331	19
Longest sequence (Mb)	2.4	7.5	5.1	10.8	8.2
% BUSCO Completeness[2]	97.6	98.6	99.5	99.4	93.8
% Repeat	57.8	46.9	2.6[3]	67.1[3]	58.9[3]
% G+C	35.7	34.5	38.2	39.8	40.7

[1] Pame, *Periplaneta americana*; Bger, *Blattella germanica*; Znev, *Zootermopsis nevadensis*; Mnat, *Macrotermes natalensis*; Lmig, *Locusta migratoria.*

[2] Extensive estimation of completeness is shown in **Supplementary Table 3**.

[3] These values were directly adapted as described in Reference 1-3.

Supplementary Table 3 Quality control of the *P. americana* assembly

Independent sequence source	#Total used entries	Recovered by the assembly	
		#Total recovered counts	%Median ratio for each entry
Clustered long transcriptome reads (PacBio)	91,421	90,782 (99.3%)	99.9
Assembled transcripts by Illumina sequencing	130,313	130,128 (99.9%)	100
Downloaded ESTs from NCBI	5,315	5,185 (97.6%)	83.5
Downloaded CDSs from GenBank	327	324 (99.1%)	96.1
CEGMA conserved proteins (*Drosophila*)	458	457 (99.8%)	90.0

Supplementary Table 4 Common features and quality control of gene sets

	Pame[1]	Bger[1]	Znev[1]	Mnat[1]	Lmig[1]
#Protein-coding genes	21,336	28,141[2]	15,860	16,310	17,586
% Coding	0.88	1.78	4.10	1.49	0.29
#Single-exon genes	2704	7984	2953	6368	3163
Med. transcript length	987	837	891	657	786
Med. exons per gene	4	2	4	2	4
Med. intron length	3005	1422	736	1480	6032
Med. transcript length	987	837	891	657	786
Completeness estimation					
% BUSCO complete	91.2	77.0	97.1	82.9	84.7
% BUSCO partial	96.4	98.4	98.7	90.4	93.6
% CEGMA recovered	98.9	99.3	99.8	98.0	96.9
% Med. coverage	97.1	91.3	97.7	94.4	96.7

[1]Pame, *Periplaneta americana*; Bger, *Blattella germanica*; Znev, *Zootermopsis nevadensis*; Mnat, *Macrotermes natalensis*; Lmig, *Locusta migratoria.*

[2]This count refers to the version 0.5.3 that we used for automated analyses in this study, which was frozen on May 2017. In a later version, v0.6.2, the total gene number was slightly changed to 28774 (ref.4).

Supplementary Table 5 Annotation of *P. americana* gene set

	# Genes with hits	% Ratio of all genes (21,336)
GO terms	11,210	52.5
KEGG KO terms	12,554	58.8
IPR domains	15,312	71.8
Pfam domains	14,575	68.3
RefSeq	18,286	85.7
UniRef50	18,260	85.6
UniProt	17,240	80.8
Cockroach (Bger)	17,807	83.5
Termite (Znev)	17,470	81.9
Fly (Dmel)	14,329	67.2
Expressed at		
Any stage	20,334	95.3
Egg	19,114	89.6
Young larva	18,492	86.7
Old larva	19,381	90.8
Adult	19,158	89.8

Supplementary Table 6 Datasets of other species used in this study

Species	URL
Drosophila melanogaster	flybase.org
Danaus plexippus[ref.5]	monarchbase.umassmed.edu
Tribolium castaneum[ref.6]	www.bioinformatics.ksu.edu/BeetleBase
Apis mellifera[ref.7]	hymenopteragenome.org
Bemisia tabaci[ref.8]	www.whiteflygenomics.org
Rhodnius prolixus[ref.9]	www.vectorbase.org
Pediculus humanus[ref,10]	www.vectorbase.org
Zootermopsis nevadensis[ref.1]	www.termitegenome.org
Macrotermes natalensis[ref.2]	gigadb.org/dataset/100057
Blattella germanica[ref.4]	NCBI#JPZV00000000
	ftp.hgsc.bcm.edu
Locusta migratoria[ref.3]	159.226.67.243

Supplementary Table 7 Top 20 expanded ortholog families in *P. Americana*

	Group	#Pame	*D*[1]	Annotation
1	MCL10012	328	121	kinesin
2	MCL10154	99	97	ankyrin
3	MCL10013	147	76	Cuticle protein
4	MCL10002	205	59	P450-CYP3 CYP4
5	MCL10312	45	44	Transcription initiation factor IIE subunit beta
6	MCL10088	90	42	ionotropic glutamate receptor
7	MCL10227	56	42	MFS transporter
8	MCL10019	96	40	Alpha-tocopherol transfer protein-like
9	MCL10408	41	39	myosin IX
10	MCL10187	56	30	Odorant receptor
11	MCL10024	87	29	Juvenile hormone-inducible protein
12	MCL10085	81	29	Ionotropic receptor
13	MCL10026	71	27	Hemolymph juvenile hormone binding protein
14	MCL10055	53	21	Nose resistant to fluoxetine protein 6
15	MCL10646	22	21	Vacuolar protein sorting-associated protein 52-like protein
16	MCL10078	89	20	Lectin-related protein
17	MCL10077	42	18	Lipase
18	MCL11673	17	17	Dopamine receptor
19	MCL10073	35	14	Elongation of very long chain fatty acids protein
20	MCL10640	19	14	zinc finger MYM-type protein 1-like

[1]*D* value indicates the difference of the number of genes in *P. americana* and the maximum value of those in all other 11 species.

Supplementary Table 8 Significantly enriched pathways (hypergeometric test) for *P. americana* genes that are more closely genetically related with the termite (*Z. nevadensis*)

Pathway[1]	Number	P-value	Q-value
RNA transport	68	2.11E-06	0.000308
Ribosome biogenesis in eukaryotes	49	2.03E-06	0.000308
Nucleotide excision repair	21	1.98E-05	0.001927
Homologous recombination	15	0.000407	0.019823
Apoptosis	15	0.000407	0.019823
DNA replication	18	0.000947	0.039489
Base excision repair	14	0.002473	0.090256
Protein processing in endoplasmic reticulum	42	0.004642	0.112952
Cell cycle	33	0.004515	0.112952
RIG-I-like receptor signaling pathway	14	0.004128	0.112952
Toll-like receptor signaling pathway	16	0.00817	0.183513
Endocytosis	39	0.010089	0.196408
NF-kappa B signaling pathway	9	0.011719	0.213867
Hippo signaling pathway -fly	16	0.014066	0.228183
Fanconi anemia pathway	16	0.016634	0.25564
mTOR signaling pathway	19	0.017962	0.262241
Non-homologous end-joining	7	0.022259	0.295439
TNF signaling pathway	13	0.024123	0.306252
Mismatch repair	10	0.028494	0.34668
Cysteine and methionine metabolism	16	0.030644	0.357925

[1] pathways related with human disease were not shown here.

Supplementary Table 9 Significantly enriched pathways (hypergeometric test) for *P. americana* genes that are more closely genetically related with the cockroach (*B. germanica*)

Pathway[1]	Number	P-value	Q-value
Fc gamma R-mediated phagocytosis	7	0.006772	0.987254
Dorso-ventral axis formation	6	0.010554	0.987254
TGF-beta signaling pathway	5	0.014097	0.987254
ErbB signaling pathway	5	0.034157	0.987254
Synaptic vesicle cycle	5	0.049897	0.987254

[1] pathways related with human disease were not shown here.

Supplementary Table 10 Top expanded InterPro domains in the *P. Americana* gene set

Num	IPR ID	#Pame	D^1	Information
1	IPR000618	214	96	Insect cuticle protein
2	IPR001128	272	94	Cytochrome P450
3	IPR002401	188	94	Cytochrome P450, E-class, group I
4	IPR005828	172	63	General substrate transporter
5	IPR001320	186	62	Ionotropic glutamate receptor
6	IPR000276	155	36	G protein-coupled receptor, rhodopsin-like
7	IPR001304	102	34	C-type lectin
8	IPR010562	82	32	Hemolymph juvenile hormone binding
9	IPR012132	63	18	Glucose-methanol-choline oxidoreductase
10	IPR011701	110	17	Major facilitator superfamily
11	IPR002172	46	11	Low-density lipoprotein (LDL) receptor class A repeat
12	IPR002213	83	10	UDP-glucuronosyl/UDP-glucosyltransferase
13	IPR010987	61	10	Glutathione S-transferase, C-terminal-like
14	IPR003663	45	9	Sugar/inositol transporter
15	IPR005821	48	9	Ion transport domain
16	IPR002018	120	8	Carboxylesterase, type B
17	IPR005804	22	7	Fatty acid desaturase, type 1
18	IPR005817	18	7	Wnt
19	IPR013098	104	7	Immunoglobulin I-set
20	IPR000074	8	6	Apolipoprotein A/E

[1]*D* value indicates the difference of the number of genes in *P. americana* and the maximum value of those in all other 11 species.

Supplementary Table 11 The gene list of TOLL pathway

	Pame[1] ID	Bger[2] ID	Dmel[3] ID	Supplementary notes or annotation
**Ligands and secreted factors**				
PGRP-SA	PaOGS34041	Bger_03988	NP_572727	Peptidoglycan recognition protein SA
PGRP-SD	PaOGS17577	Bger_21009	NP_648145	Peptidoglycan recognition protein SD
GNBP1	PaOGS26893	Bger_06164	NP_524142	Gram-negative bacteria binding protein 1
	PaOGS26894	Bger_27821		
	PaOGS17659	Bger_06148		
	PaOGS28939	Bger_27120		
	PaOGS16088	Bger_25956		
	PaOGS04407			
	PaOGS28938			
	PaOGS16087			
	PaOGS15533			
	PaOGS25933			
	PaOGS25932			
	PaOGS25181			
GNBP2	ND	ND	NP_524141	Gram-negative bacteria binding protein 2
GNBP3	PaOGS36768	Bger_00528	NP_523986	Gram-negative bacteria binding protein 3
	PaOGS12546	Bger_25957		
ModSP	PaOGS18899	Bger_06353	NP_536776	modular serine protease
Grass	ND	ND	NP_651543	Gram-positive Specific Serine protease
Spirit	ND	ND	NP_572492	Serine Protease Immune Response Integrator

Spz	PaOGS23351<br>PaOGS15255<br>PaOGS36598	Bger_09349<br>Bger_09350	NP_524526	Spatzle
Spz2	PaOGS34707<br>PaOGS06743	Bger_25455	NP_729009	Neurotrophin 1, NT1, dNT1, CG18318
Spz3	PaOGS32088	Bger_14948	NP_609160	spatzle 3
Spz4	PaOGS29033	Bger_01655	NP_609504	spatzle 4
Spz5	PaOGS14320<br>PaOGS15255<br>PaOGS00122	Bger_09350	NP_647753	spatzle 5, DNT2, NT2
easter	PaOGS18716<br>PaOGS07008<br>PaOGS20874	Bger_09760<br>Bger_09770	NP_524362	ea
SPE	PaOGS18715	Bger_09757	NP_651168	Spatzle-Processing Enzyme
**Receptor**				
Toll	PaOGS03981<br>PaOGS30622	ND	NP_001262995	T1
Toll-2	PaOGS02723	Bger_10640	NP_476814	18 wheeler
Toll-3	PaOGS29429	Bger_28181	NP_649719	MstProx, dTLR3
Toll-4	ND	ND	NP_523519	dTLR4, Toll-like
Toll-5	ND	ND	NP_477438	Tehao, Dtlr5
Toll-6	PaOGS30981<br>PaOGS05166	Bger_24515<br>Bger_27153	NP_524081	dTLR6
Toll-7	PaOGS22033	Bger_28416	NP_523797	dTLR7
Toll-8	PaOGS30622<br>PaOGS37362	Bger_20008<br>Bger_02076	NP_524757	Tollo, Dtlr8

Toll-9	PaOGS28306<br>PaOGS00449<br>PaOGS29874<br>PaOGS29876<br>PaOGS01025	Bger_03247<br>Bger_16712	NP_649214	
**Signal transduction pathway**				
MyD88 (symbol "9" Figure 3)	PaOGS03951	Bger_09614	NP_610479	dMyd88, DmMyD88, kra, krapfen
Tube (symbol "10" Figure 3)	ND	ND	NP_001189164	
pelle	PaOGS05858<br>PaOGS12548	Bger_18970<br>Bger_20734	NP_476971	pll
Pellino	PaOGS18538<br>PaOGS32350<br>PaOGS18537	Bger_11668<br>Bger_19629<br>Bger_25292	NP_524466	Pli
Cactus	PaOGS13946<br>PaOGS33742<br>PaOGS31963	Bger_01858<br>Bger_06011	NP_723960	cact
Dorsal	PaOGS14847	Bger_22543	NP_724054	dl
Deaf1	PaOGS36637	Bger_24394	NP_524169	Deformed epidermal autoregulatory factor-1
dif*	ND	ND	NP_001162998	Dorsal-related immunity factor
Gprk2*	PaOGS00819	Bger_20197	NP_476867	G protein-coupled receptor kinase 2

[1] Pame, *Periplaneta americana*, [2] Bger, *Blattella germanica*, [3] Dmel, *Drosophila melanogaster*. * = gene not shown in the Figure 3a because it's not in the main pathway.

Supplementary Table 12 The gene list of IMD pathway

	Pame[1] ID	Bger[2] ID	Dmel[3] ID	Supplementary notes or annotation
**Ligands and extracellular proteins**				
PGRP-SB1	PaOGS09251	Bger_21011	NP_648917	Peptidoglycan recognition protein SB1
PGRP-SB2	PaOGS12466	Bger_07207	NP_001261970	Peptidoglycan recognition protein SB2
PGRP-SC1a	PaOGS08236	Bger_12586	NP_610407	Peptidoglycan recognition protein SC1a
PGRP-SC1b	ND	ND	NP_610409	Peptidoglycan recognition protein SC1b
PGRP-SC2	PaOGS00830	Bger_12080	NP_610410	Peptidoglycan recognition protein SC2
PGRP-LB	PaOGS35371<br>PaOGS19147<br>PaOGS19148<br>PaOGS10768<br>PaOGS35369<br>PaOGS00826<br>PaOGS17579<br>PaOGS15599	Bger_07207<br>Bger_07208	NP_001247052	Peptidoglycan recognition protein LB
PGRP-LE	PaOGS25508	Bger_21009	NP_573078	Peptidoglycan recognition protein LE
PGRP-LF	PaOGS28006<br>PaOGS01447	Bger_21010<br>Bger_12586<br>Bger_15019	NP_648299	Peptidoglycan recognition protein LF

**Receptor**				
PGRP-LC	PaOGS28008	Bger_21011	NP_648298	Peptidoglycan recognition protein LC
**Signal transduction pathway**				
imd	PaOGS00913	ND	NP_573394	immune deficiency
Pirk	PaOGS00402	Bger_00547	NP_611598	poor Imd response upon knock-in
FADD	PaOGS28864	Bger_00847	NP_651006	Fas-associated death domain
DREDD	PaOGS31167	Bger_14092	NP_477249	Death related ced-3/Nedd2-like caspase
Dnr1	PaOGS16033	Bger_09439	NP_001261137	defense repressor 1
Relish	PaOGS33475	Bger_13050	NP_477094	Rel, NF-κB, ird4
caspar	PaOGS04016	Bger_10382	NP_611080	casp
Sickie	PaOGS25134	Bger_19906	NP_001260606	sick
	PaOGS15258	Bger_27334		
ben	PaOGS21776	Bger_14002	NP_511150	bendless，Ubc13, UbcD3
	PaOGS19014	Bger_18381		
Effete	PaOGS20997	Bger_12667	NP_731941	Effete, UbcD1, UbcD, E(Pc)88D, UBC4, ms(3)88D
TAK1	PaOGS13569	Bger_05441	NP_524080	TGF-β activated kinase 1
Tab2	PaOGS00384	Bger_07566	NP_611408	TAK1-associated binding protein 2
Key	PaOGS01862	Bger_22316	NP_523856	kenny,IKKγ, DmIKKγ, Kenny, IKK, IKKg
Ird5	PaOGS10933	Bger_00233	NP_524751	I-kappaB kinase β, DmIkkβ, IKKβ, IKKb, IRD-5, IKK
kay	PaOGS04497	Bger_03487	NP_001027577	kayak,Fos, DFos, D-Fos, AP-1, dFRA, CG15507, CG15509, c-Fos, AP1, fra
Stat92E	PaOGS14791	Bger_06093	NP_001262760	Signal-transducer and activator of transcription protein at 92E
	PaOGS11542	Bger_19443		
Iap2	PaOGS03160	Bger_00682	NP_477127	Death-associated inhibitor of apoptosis 2

	PaOGS03203	Bger_09439		also known as D-IAP2, DIAP-2, DIHA
	PaOGS05706	Bger_01768		
	PaOGS16033	Bger_01770		
	PaOGS17338	Bger_14494		
	PaOGS21965	Bger_11620		
	PaOGS29236	Bger_15389		
	PaOGS31717	Bger_20862		
	PaOGS32827			
Akirin*	PaOGS28957	Bger_14274	NP_648113	bhr
HDAC1*	PaOGS04186	Bger_01952	NP_647918	Histone deacetylase 1, rpd3, dHDAC1, DRpd3, HDAC, l(3)04556, Su(var)326
Bsk*	PaOGS14087	Bger_26268	NP_723541	JNK, DJNK, D-JNK, pJNK, basket

[1] Pame, *Periplaneta americana*, [2] Bger, *Blattella germanica*, [3] Dmel, *Drosophila melanogaster*. * = gene not shown in the Figure 3a because it's not in the main pathway.

Supplementary Table 13 The gene list of JAK-STAT pathway

	Pame[1] ID	Bger[2] ID	Dmel[3] ID	Supplementary notes or annotation
**Ligands and secreted factors**				
Upd1	ND	ND	NP_525095	unpaired 1
Upd2	ND	ND	NP_573277	unpaired 2
Upd3	ND	ND	NP_001097014	unpaired 3
**Receptor**				
dome	PaOGS14246	Bger_01267	NP_523412	domeless
**Signal transduction pathway**				
Hop	PaOGS29402	Bger_18756	NP_511119	Jak, hopscotch
Stat92E	PaOGS14791	Bger_06093	NP_001262760	Signal-transducer and activator of transcription protein at 92E.
PIAS	PaOGS36496	Bger_11142	NP_724749	Suppressor of variegation 2-10
RanBP3	PaOGS32875	Bger_12731	NP_651178	Ran binding protein 3
Ptp61F	PaOGS11757	Bger_26148	NP_476687	Protein tyrosine phosphatase 61F
Socs36E	PaOGS24594	Bger_05239	NP_523593	Suppressor of cytokine signaling at 36E
ken	PaOGS26381	Bger_23824	NP_523833	ken and barbie
Cnot4	PaOGS20210	Bger_01413	NP_001260343	CCR4-NOT transcription complex subunit 4, NOT4
EloB*	PaOGS25195	Bger_23739	NP_524416	Elongin B
EloC*	PaOGS05017	Bger_25099	NP_725894	Elongin C
Cullin 5*	PaOGS28614	Bger_07822	NP_651665	cul-5, CUL5

[1] Pame, *Periplaneta americana*, [2] Bger, *Blattella germanica*, [3] Dmel, *Drosophila melanogaster*. * = gene not shown in the Figure 3a because it's not in the main pathway.

Supplementary Table 14 The gene list of PPO and its associated genes

	Pame[1] ID	Bger[2] ID	Dmel[3] ID	Supplementary notes or annotation
**Sp7**	ND	ND	NP_649734	Serine protease 7, MP2, PAE1
**Spn27A**	PaOGS25165	Bger_11362	NP_652024	Serpin 27A
**Spn28Dc**	PaOGS02715	Bger_24516<br>Bger_04527	NP_609172	Serpin 28Dc, spn28D
**Spn77Ba**	PaOGS01312	Bger_15289	NP_649205	Serpin 77Ba
**Hayan**	ND	ND	NP_001097020	
**MP1**	PaOGS14968<br>PaOGS20874<br>PaOGS27348<br>PaOGS18714	Bger_09767<br>Bger_09770<br>Bger_09757	NP_001303421	Melanization Protease 1
**PPO1**	ND	ND	NP_476812	Prophenoloxidase 1
**PPO2**	PaOGS19761<br>PaOGS19762	Bger_20290<br>Bger_20291	NP_610443	Prophenoloxidase 2
**PPO3**	PaOGS33075<br>PaOGS33082	Bger_00852	NP_524760	Prophenoloxidase 3

[1] Pame, *Periplaneta americana*, [2] Bger, *Blattella germanica*, [3] Dmel, *Drosophila melanogaster*.

Supplementary Table 15 The gene list of AMPs

	Pame[1] ID	Bger[2] ID	Dmel[3] ID	Supplementary notes or annotation
**AFP**	PaOGS35945	ND	ND	
**Attacin-A**	PaOGS36940	ND	NP_523745	
**Defensin**	PaOGS22104	Bger_22590	NP_523672	
	PaOGS22105	Bger_24006		
	PaOGS25963			
	PaOGS25964			
	PaOGS25965			
**Drosomycin**	PaSCF26382 17981—18112	Bger_12810	NP_523901	
**Pro-rich peptides**	PaSCF10328 901670----901774	ND	ND	
**termicin**	PaSCF17378 12249-12097(termicin-1）	Bger_00758	ND	
	PaSCF17378 43696-43598(termicin-2)	Bger_26761		
		Bger_26762		

[1] Pame, *Periplaneta americana*, [2] Bger, *Blattella germanica*, [3] Dmel, *Drosophila melanogaster*.

Supplementary Table 16 The gene list of 20E pathway

	Pame[1] ID	Bger[2] ID	Dmel[3] ID	Supplementary notes or annotation
**Ecdysone receptor**				
**EcR**	PaOGS33228	Bger_08790	NP_724460	ecdysone receptor
**USP**	PaOGS21233	Bger_15477	NP_476781	ecdysone receptor
	PaOGS36136			
**20E primary response gene**				
**E75**	PaOGS33408	Bger_26014	NP_730321	ecdysone-induced protein
**E74**	PaOGS20118	Bger_22825	NP_730288	ecdysone-induced protein
	PaOGS31546	Bger_11041		
**Br-C**	PaOGS00309	Bger_00841	NP_001162638	ecdysone-induced protein
	PaOGS02465	Bger_03570		
	PaOGS05966	Bger_03579		
	PaOGS17094	Bger_04067		
	PaOGS17458	Bger_04233		
	PaOGS24449	Bger_06368		
	PaOGS28676	Bger_12704		
	PaOGS31102	Bger_15807		
	PaOGS32246	Bger_15950		
	PaOGS34612	Bger_18118		
	PaOGS34937	Bger_22239		
		Bger_22365		

		Bger_26295<br>Bger_27201		
**E93**	PaOGS00579	Bger_11783<br>Bger_22028	NP_001262817	ecdysone-induced protein
**Hr3**	PaOGS31710	Bger_00728	NP_001334718	steroid hormone mediated signaling pathway
**Ftz-F1**	PaOGS36905<br>PaOGS36906	Bger_09988	NP_524143	steroid hormone mediated signaling pathway
**Ecdysone biosynthesis and biodegradation**				
**Nvd**	PaOGS33111	Bger_22030	NP_001097670	Converts cholesterol to 7-dehydrocholesterol
**Spo/Spok**	PaOGS14459<br>PaOGS34772<br>PaOGS28150	Bger_04901<br>Bger_25648	NP_647975	Converts 7-dehydrochoresterol to 2,22,25-trideoxyecdysone(ketodiol)
**Phm**	PaOGS32253<br>PaOGS25093	Bger_21433	NP_573319	Converts 2,22,25-trideoxyecdysone (ketodiol) to 22,2-dideoxyecdysone (ketotriol)
**Dib**	PaOGS32929	Bger_24331	NP_524810	Converts 22,2-dideoxyecdysone (ketotriol) to 2-deoxyecdysone
**Sad**	PaOGS03950	Bger_09617	NP_650123	Converts 2-deoxyecdysone to α-ecdysone (E)
**Shd**	PaOGS36055<br>PaOGS08749	Bger_13798	NP_996074	Converts α-ecdysone (E) to 20-hydroxyecdysone (20E)
**CYP18A1**	PaOGS25100<br>PaOGS28912<br>PaOGS03819	Bger_09756<br>Bger_09764<br>Bger_09765	NP_728191	inactivates 20-hydroxyecdysone

[1] Pame, *Periplaneta americana*, [2] Bger, *Blattella germanica*, [3] Dmel, *Drosophila melanogaster*.

Supplementary Table 17 The gene list of JH pathway

	Pame[1] ID	Bger[2] ID	Dmel[3] ID	Supplementary notes or annotation
**JH receptor**				
**Met**	ND	ND	NP_511126	
**GCE**	PaOGS20531	ND	NP_001259564	JH receptor
**JH response gene**				
**Kr-h1**	PaOGS24593<br>PaOGS35342	Bger_08591	NP_477466	JH primary response gene
**Hsp83**	PaOGS00318	Bger_03740<br>Bger_05508	NP_523899	unfolded protein binding
**JH biosynthesis and biodegradation**				
**FAMeT**	PaOGS28839<br>PaOGS08986<br>PaOGS32140	Bger_20349<br>Bger_20710	NP_611544	Farnesoic acid O-methyl transferase
**JHEH**	PaOGS12138<br>PaOGS16223<br>PaOGS22049	Bger_06817<br>Bger_06819	NP_611385	juvenile hormone epoxide hydrolase
**HMGS**	PaOGS31743<br>PaOGS37360	Bger_02739<br>Bger_03932	NP_524711	HMG Coenzyme A synthase
**HMGR**	PaOGS01132	Bger_14007	NP_732900	hydroxymethylglutaryl-CoA reductase (NADPH) activity

	PaOGS15601			
**IPP**	PaOGS02335	Bger_04718	NP_731872	iNDsitol-1,4-bisphosphate 1-phosphatase activity
	PaOGS04500	Bger_03482		
	PaOGS20547	Bger_14147		
**FPPS**	PaOGS04443	Bger_01636	NP_477380	geranyltranstransferase activity
	PaOGS04442	Bger_01637		
	PaOGS18036			
	PaOGS03620			
	PaOGS03618			
	PaOGS03619			
	PaOGS10848			
	PaOGS16681			
	PaOGS03621			
**JHAMT**	PaOGS26372	Bger_04116	NP_609793	juvenile hormone acid methyltransferase activity
	PaOGS26016			
	PaOGS20267			
	PaOGS26373			
	PaOGS35164			
	PaOGS20304			
	PaOGS23365			
	PaOGS06349			
	PaOGS12062			
	PaOGS23367			
	PaOGS27228			
	PaOGS23363			
	PaOGS23364			

**CYP15A1**	PaOGS36907	Bger_09987	AAN06321	Methyl farnesoate epoxidase
**AACT**	PaOGS05420<br>PaOGS21968<br>PaOGS24518<br>PaOGS26519	Bger_01766<br>Bger_10151	NP_572414	acetyl-CoA C-acetyltransferase activity
**mevK**	PaOGS12817<br>PaOGS12818	Bger_05361	NP_001027412	mevalonate kinase activity
**pmevK**	ND	ND	ND	
**DPMD**	ND	ND	ND	
**IPPI**	PaOGS20501	Bger_04562	NP_650962	isopentenyl-diphosphate delta-isomerase activity
**FPPase**	PaOGS20286<br>PaOGS33070<br>PaOGS34252	Bger_04095<br>Bger_14027	NP_572760	alkaline phosphatase activity
**Fod(ADH)**	PaOGS02065<br>PaOGS06921<br>PaOGS10983<br>PaOGS10984<br>PaOGS15149<br>PaOGS17310<br>PaOGS23408<br>PaOGS28284<br>PaOGS28285<br>PaOGS28357	Bger_05936<br>Bger_05937<br>Bger_05941<br>Bger_07328<br>Bger_07356<br>Bger_22502	NP_001027266	acetaldehyde dehydrogenase (acetylating) activity
**Fad (ALDH)**	PaOGS14229	Bger_00783	NP_609285	aldehyde dehydrogenase (NAD) activity

	PaOGS28787	Bger_02689		
	PaOGS18382	Bger_03771		
	PaOGS30290	Bger_05316		
	PaOGS10653	Bger_13049		
	PaOGS18875	Bger_14856		
		Bger_15688		
		Bger_18207		
**JHE**	PaOGS11091	Bger_00414	NP_523758	carboxylic ester hydrolase activity; juvenile-hormone esterase activity
	PaOGS16012	Bger_10745		
	PaOGS16015	Bger_18826		
	PaOGS16014			
	PaOGS03076			
**JHDK**	PaOGS06270	Bger_02790	NP_524381	sarcoplasmic calcium-binding protein 2

[1] Pame, *Periplaneta americana*, [2] Bger, *Blattella germanica*, [3] Dmel, *Drosophila melanogaster*.

Supplementary Table 18 The gene list of insulin pathway

	Pame[1] ID	Bger[2] ID	Dmel[3] ID	Supplementary notes or annotation
**Ligands and secreted factors**				
convoluted	PaOGS29089	Bger_24097	NP_610948	carboxypeptidase N regulatory subunit
	PaOGS24452	Bger_15284		
	PaOGS25673	Bger_19693		
Ecdysone-inducible gene L2	ND	ND	NP_523921	insulin-related peptide binding protein
Insulin-like peptide	PaOGS15477	Bger_24050	NP_648359	Insulin-like peptide 1
	PaOGS02783		NP_524012	Insulin-like peptide 2
	PaOGS02784		NP_648360	Insulin-like peptide 3
	PaOGS15476		NP_648361	Insulin-like peptide 4
	PaOGS23100		NP_996037	Insulin-like peptide 5
	PaOGS36319		NP_570000	Insulin-like peptide 6
			NP_570070	Insulin-like peptide 7
Short neuropeptide-F	PaOGS17125	Bger_20322	NP_524176	short neuropeptide F receptor
	PaOGS09479	Bger_20131		
IGF-II mRNA-binding protein	PaOGS03214	Bger_18295	NP_001285091	Insulin-like growth factor 2 mRNA-binding protein
**Insulin-like receptor and its substrates**				
chico	PaOGS37252	Bger_01864	NP_723540	insulin receptor substrate-1
		Bger_01863		

Insulin-like receptor	PaOGS10688	Bger_10316	AAF55903	insulin receptor
	PaOGS25540	Bger_13643		similar to Insulin receptor precursor
	PaOGS25539	Bger_13644		
	PaOGS31689	Bger_14014		
	PaOGS03239			
	PaOGS36621			
	PaOGS02593			No significant similarity found
Pi3K92E	PaOGS25629	Bger_10044	NP_650902	Phosphotidylinositol 3 kinase 92E
Pi3K21B	PaOGS11039	Bger_15654	NP_477270	phosphatidylinositol 3-kinase 21B
	PaOGS26305			
Pi3K68D	PaOGS21297	Bger_01070	NP_001163420	Phosphotidylinositol 3 kinase 68D
	PaOGS04183			
	PaOGS04184			
	PaOGS21298			
Pi3K59F	PaOGS06680	Bger_06697	NP_477133	phosphotidylinositol 3 kinase 59F
**Signal transduction pathway**				
Akt1	PaOGS21842	Bger_07332	NP_732114	serine/threonine protein kinase Akt
focal adhesion kinase	PaOGS08207	Bger_02892	NP_523793	Focal adhesion kinase 1
melted	PaOGS08596	Bger_19955	NP_001014572	melted
nucleostemin 3	PaOGS36409	Bger_02421	NP_569915	Large subunit GTPase 1-like protein
	PaOGS14310			
SCYLLA	PaOGS35498	Bger_01043	AAF59841	similar to charybde
CHARYBDE	PaOGS35498	Bger_01043	AAF59840	similar to charybde
target of rapamycin	PaOGS00312	Bger_07577	NP_524891	target of rapamycin isoform 1
	PaOGS16261	Bger_27083		
	PaOGS28942			

Tsc1	PaOGS22671	Bger_08706	NP_477415	Tsc1
twins	PaOGS22334	Bger_15088	NP_476880	phosphatase pp2a or twins
widerborst	PaOGS24441	Bger_07755	NP_733215	similar to widerborst-PC
PP2A-B'	PaOGS25786<br>PaOGS10864	Bger_00705	NP_001262695	PP2A 56 kDa regulatory subunit delta isoform
Myc	PaOGS28552	Bger_11276	NP_525062	Myc protein
forkhead box, sub-group O	PaOGS31435	Bger_24290	NP_996205	similar to forkhead box subgroup O
gigas(TSC2)	PaOGS25191	Bger_09893	NP_524177	similar to tuberous sclerosis 2
ribosomal protein S6	PaOGS26661<br>PaOGS02013	Bger_02990	NP_727213	ribosomal protein S6
spargel	PaOGS23091	ND	NP_730835	spargel
4E-BP1（thor）	PaOGS17099	Bger_22986	NP_477295	eukaryotic translation initiation factor 4E-binding protein 1
AMPKalpha	PaOGS22353	Bger_16490	NP_477313	5'-AMP-activated protein kinase, catalytic alpha subunit
AMPKbeta	PaOGS21039	Bger_07371	NP_610460	5'-AMP-activated protein kinase, regulatory beta subunit
AMPKgama	PaOGS34091	Bger_18283	NP_001163672	5'-AMP-activated protein kinase, regulatory gamma subunit
CAP	PaOGS35184<br>PaOGS35183<br>PaOGS10470	Bger_04936<br>Bger_04937<br>Bger_00872<br>Bger_04938	NP_001246239	sorbin and SH3 domain-containing protein 1
caravaggio	ND	ND	NP_524477	Mitochondrial 28S ribosomal protein S31
Cbl	PaOGS25958	Bger_02640<br>Bger_02641	CAA11149	E3 ubiquitin-protein ligase CBL
nejire	PaOGS25711<br>PaOGS23031<br>PaOGS35225	Bger_12712<br>Bger_12714<br>Bger_12715	NP_001188575	E1A/CREB-binding protein

		Bger_22304		
Cip4	PaOGS19358<br>PaOGS19357	Bger_13506	NP_647893	Cdc42-interacting protein 4
Raf	PaOGS36071	Bger_28322	NP_525047	Raf-like protein serine/threonine-protein kinase phl
Crk	PaOGS30787	Bger_09259	NP_726549	proto-oncogene C-crk
Ehbp1	PaOGS20136	Bger_14504	NP_611179	Eps15 homology domain containing protein-binding protein 1
ecdysone receptor	PaOGS33228	Bger_08790	NP_001163061	Ecdysone receptor
Past1	PaOGS16748<br>PaOGS22627<br>PaOGS03852	Bger_15881<br>Bger_22702	NP_524332	Putative Achaete Scute Target 1
eIF-4E	PaOGS28859<br>PaOGS29690<br>PaOGS15419	Bger_00848<br>Bger_00898<br>Bger_07664	NP_729485	translation initiation factor 4E
ppk	PaOGS00759<br>PaOGS08174<br>PaOGS09663<br>PaOGS09884<br>PaOGS10495<br>PaOGS12339<br>PaOGS15212<br>PaOGS15950<br>PaOGS18067<br>PaOGS20205<br>PaOGS25907<br>PaOGS26103	Bger_00502<br>Bger_00506<br>Bger_00509<br>Bger_01660<br>Bger_03023<br>Bger_05822<br>Bger_07055<br>Bger_16722<br>Bger_18015	NP_477232	Sodium channel protein Nach OS

	PaOGS28784<br>PaOGS29037<br>PaOGS30636<br>PaOGS31329<br>PaOGS33388<br>PaOGS33919<br>PaOGS34603<br>PaOGS34638<br>PaOGS36017			
Erk(rolled)	PaOGS23998<br>PaOGS05015	Bger_07900	NP_001287635	Mitogen-activated protein kinase
flotillin 1	PaOGS15301<br>PaOGS36285	Bger_07684	NP_725476	Flotillin-1
Glut4EF	PaOGS34976	Bger_11307	ABY40732	Glucose transporter 4 enhancer factor
pico	PaOGS35141	Bger_10233	NP_608363	Ras-associated and pleckstrin-like proteiny domains-containing protein 1
drk	PaOGS19560	Bger_03191	NP_725306	downstream of receptor kinase
GS	PaOGS16444<br>PaOGS01368	Bger_09447	NP_001162783	Glutathione Synthetase
shaggy	PaOGS21420	Bger_15050<br>Bger_15051	NP_001259193	glycogen synthase kinase-3
Hormone-sensitive lipase	PaOGS13893	ND	NP_725942	Hormone-sensitive lipase is involved in lipid storage
I-kappaB kinase epsilon	PaOGS03122	Bger_23021	NP_610048	Serine/threonine-protein kinase TBK1

inos	PaOGS04139	Bger_04317	NP_477405	Inositol-3-phosphate synthase 1-B
basket	PaOGS14087	Bger_26268	NP_001162932	Stress-activated protein kinase JNK
Lkb1 kinase	PaOGS07226	Bger_01519	NP_001163606	serine/threonine-protein kinase 11
Dsor1	PaOGS14031	Bger_23552	BAA02925	dual specificity mitogen-activated protein kinase dSOR1
dreadlocks	PaOGS03985<br>PaOGS21016	Bger_16202<br>Bger_27896	NP_001259845	cytoplasmic protein NCK1
phosphatase and tensin homolog	PaOGS02078<br>PaOGS21433	Bger_02874<br>Bger_19611	NP_599147	phosphatase and tensin-like
Phosphoinositide-dependent kinase 1	PaOGS02814	Bger_19061	NP_001261183	3-phosphoinositide-dependent protein kinase 1 isoform X1
RPS6-p70-protein kinase	PaOGS28537<br>PaOGS34494<br>PaOGS32255	Bger_22784<br>Bger_22785<br>Bger_27047<br>Bger_27124<br>Bger_23850<br>Bger_25546	NP_523941	ribosomal protein S6 kinase
protein kinase, cAMP-dependent, catalytic subunit 1	PaOGS33117	Bger_01349	NP_995672	protein kinase A
inactivation no afterpotential C	PaOGS08405<br>PaOGS02145<br>PaOGS04744<br>PaOGS13125	Bger_00689<br>Bger_16364<br>Bger_20742<br>Bger_27207	NP_476863	PKC (protein kinase C)
protein phosphatase 1alpha at 96A	PaOGS07811<br>PaOGS01282<br>PaOGS26177	Bger_01574	NP_001262919	protein phosphatase 1, catalytic subunit

	PaOGS11408			
microtubule star	PaOGS27589	Bger_23717<br>Bger_25259	NP_001285725	protein phosphatase 2 (formerly 2A), catalytic subunit
protein phosphatase V	PaOGS31508	Bger_18449	NP_001259274	serine/threonine-protein phosphatase 6 catalytic subunit
Proline-rich Akt substrate 40 kDa	ND	ND	NP_001303349	
protein tyrosine phosphatase 61F	PaOGS11757	Bger_26148	NP_001261253	protein tyrosine phosphatase, non-receptor type 1
Rac1	PaOGS23910	Bger_09087	NP_476950	Ras-related C3 botulinum toxin substrate 1
Ras 85D	PaOGS07457<br>PaOGS02503	Bger_01384<br>Bger_06705	NP_476699	Ras-like protein
Ras 64B	PaOGS15449<br>PaOGS04602	Bger_00921<br>Bger_27659<br>Bger_14904	NP_523917	Ras-related protein R-Ras2
Rheb	PaOGS37079<br>PaOGS01402<br>PaOGS01401	Bger_14087	NP_730951	Ras homolog enriched in brain
triose phosphate isomerase	PaOGS10090<br>PaOGS32192<br>PaOGS35207	Bger_11437	NP_788764	triosephosphate isomerase
SHC-adaptor protein	PaOGS31868	Bger_09874	NP_524683	src homology 2 domain-containing transforming protein C
corkscrew	PaOGS29953<br>PaOGS02284<br>PaOGS29346	Bger_09096	NP_001259174	protein tyrosine phosphatase, non-receptor type 11
Salt-inducible kinase 2	PaOGS27630	Bger_16996<br>Bger_19023	NP_569972	serine/threonine-protein kinase NIM1, putative
SAPK-interacting protein 1	PaOGS13689	Bger_14252	NP_610963	Stress-activated map kinase-interacting protein 1
Son of sevenless	PaOGS26097	Bger_12100	NP_476597	as GTP exchange factor, son of sevenless, putative

	PaOGS37301	Bger_16633		Ras-specific guanine nucleotide-releasing factor 2
	PaOGS05185			
sterol regulatory element binding protein	PaOGS15611	Bger_16229	NP_524166	sterol regulatory element-binding transcription factor 1
pollux	PaOGS20378	ND	NP_001163511	TBC1 domain family member 4 isoform X2
	PaOGS01110			TBC1 domain family member 1 isoform X3
C3G guanyl-nucleotide exchange factor	PaOGS31746	Bger_03933	NP_001259293	Rap guanine nucleotide exchange factor (GEF) 1
	PaOGS01706			
Usf	PaOGS13899	Bger_18713	NP_726931	Upstream stimulatory factor, putative
USP	PaOGS21233	Bger_15477	NP_476781	ultraspiracle
	PaOGS36136			

[1] Pame, *Periplaneta americana*, [2] Bger, *Blattella germanica*, [3] Dmel, *Drosophila melanogaster*. * = potential fragments of one gene, respectively

Supplementary Table 19 The gene list of Chitin metabolism pathway

	Pame[1] ID	Bger[2] ID	Dmel[3] ID	Supplementary notes or annotation
**Chitin syntheses**				
Trehalase	PaOGS34620	Bger_07250	CG9364	Trehalase
	PaOGS36391	Bger_24156		
	PaOGS13872*	Bger_01391		
	PaOGS20505*	Bger_14170		
	PaOGS16530			
	PaOGS02695			
Hexokinase A	PaOGS11144	Bger_12151	CG3001	Hexokinase A
	PaOGS27280	Bger_17436		
	PaOGS08108	Bger_18479		
Glucose-6-phosphate isomerase	PaOGS21361	Bger_06841	CG8251	Pgi Phosphoglucose isomerase
Glutamine: fructose-6-phosphate aminotransferase	PaOGS37424	Bger_14767	CG12449	GFAT1
	PaOGS32027		CG1345	GFAT2
	PaOGS23873			
Glucosamine-6-phosphate N-acetyltransferase	PaOGS04499	Bger_03483	CG1969	GNA
Phosphoacetylglucosamine mutase	PaOGS06368	Bger_06051	CG10627	Nesthocker, nst, (Phosphoacetylglucosamine mutase)
UDP-N-acetylglucosamine	ND	Bger_20395	CG9535	mummy

pyrophosphorylase				UDP-N-acetylglucosamine pyrophosphorylase
Chitin synthase 1	PaOGS16634	Bger_05690	CG2666	krotzkopf verkehrt, kkv (Chitin synthase 1)
		Bger_05692		KKV
Chitin synthase 2	PaOGS16634	Bger_05690	CG7464	Chitin synthase 2 (CHS2)
		Bger_05692		CHS2
**Chitin degradation**				
Imaginal disc growth factor	PaOGS29955	Bger_26630	CG4472	IDGF1
			CG4475	IDGF2
			CG4559	IDGF3
			CG1780	IDGF4
			CG5154	IDGF5
Chitinase 1	PaOGS30173	Bger_10354	CG8460	Chitinase 1
Chitinase 2	PaOGS14170	Bger_13389	CG2054	Chitinase 2
Chitinase 3	PaOGS33360	Bger_14104	ND	Chitinase 3
Chitinase 5	PaOGS07061*	Bger_02854	CG9307	Chitinase 5
	PaOGS07062*	Bger_02855		
	PaOGS07060	Bger_02856		
	PaOGS32991			
Chitinase 6	PaOGS13150	Bger_16010	CG43374	Chitinase 6
Chitinase 7	PaOGS17511	Bger_26494	CG1869	Chitinase 7
Chitinase 10	PaOGS37217	Bger_01400	CG18140	Chitinase 10
Chitinase 11	PaOGS19890	Bger_12136	CG3044	Chitinase 11
Chitinase 8 (other chitinases)	PaOGS02617	Bger_08812	CG9357	Chitinase 8
	PaOGS25829	Bger_01401	CG3986	Chitinase 4
	PaOGS04885	Bger_01402	CG10531	Chitinase 9
	PaOGS25832	Bger_14524	CG30293	Chitinase 12

	PaOGS34878
	PaOGS25831
	PaOGS09724
	PaOGS25833
	PaOGS04059

[1]Pame = *Periplaneta americana*, [2]Bger = *Blattella germanica*, [3]Dmel = *Drosophila melanogaster*. * = potential fragments of one gene, respectively

Supplementary Table 20 The gene list of AMPK pathway

	Pame[1] ID	Bger[2] ID	Dmel[3] ID	Supplementary notes or annotation
**Subunits**				
AMPKalpha	PaOGS22353	Bger_16490	NP_477313	5'-AMP-activated protein kinase, catalytic alpha subunit
AMPKbeta	PaOGS21039	Bger_07371	NP_610460	5'-AMP-activated protein kinase, regulatory beta subunit
AMPKgama	PaOGS34091	Bger_18283	NP_001163672	5'-AMP-activated protein kinase, regulatory gamma subunit
**Signal transduction pathway**				
4E-BP1（thor）	PaOGS17099	Bger_22986	NP_477295	eukaryotic translation initiation factor 4E-binding protein 1
Acetyl-CoA carboxylase	PaOGS02215	Bger_06031	NP_610342	acetyl-CoA carboxylase / biotin carboxylase
Akt1	PaOGS21842	Bger_07332	NP_732114	serine/threonine protein kinase Akt
Autophagy-related 1	PaOGS18161<br>PaOGS07965	Bger_10975	NP_001163433	Serine/threonine-protein kinase
Autophagy-related 13	PaOGS17978	Bger_11856	NP_649796	autophagy-related protein 13
Autophagy-related 17	PaOGS08238	Bger_00056	NP_649573	Autophagy-related 17
brummer	PaOGS34957	Bger_05619	NP_001163445	patatin-like phospholipase domain-containing protein 3
cytoplasmic linker protein 190	PaOGS02216	Bger_19402	NP_001137834	CAP-Gly domain-containing linker protein 1
Cyclic-AMP response element binding protein B	PaOGS09279	Bger_06395	NP_001334685	cAMP-responsive element modulator isoform X4
Erk(rolled)	PaOGS23998<br>PaOGS05015	Bger_07900	NP_001287635	Mitogen-activated protein kinase

forkhead box, sub-group O	PaOGS31435	Bger_24290	NP_996205	similar to forkhead box subgroup O
GS	PaOGS16444	Bger_09447	NP_001162783	Glutathione Synthetase
	PaOGS01368			Glutathione Synthetase
G protein alpha q subunit	PaOGS27751	Bger_17163	NP_725192	guanine nucleotide binding protein (G protein), alpha 14
	PaOGS24883	Bger_17164		
	PaOGS06393	Bger_17165		
histone deacetylase 4	PaOGS25592	Bger_13509	NP_001259507	histone deacetylase 4
HMG coenzyme A reductase	PaOGS01132	Bger_14007	NP_732900	3-hydroxy-3-methylglutaryl-coenzyme A reductase
	PaOGS15601			
hepatocyte nuclear factor 4	PaOGS26261	Bger_07640	NP_723413	Transcription factor HNF-4-like protein
Insulin-like receptor	PaOGS10688	Bger_14014	AAF55903	insulin receptor
	PaOGS25540	Bger_10316		similar to Insulin receptor precursor
	PaOGS25539	Bger_13643		
	PaOGS31689	Bger_13644		
	PaOGS36621			
	PaOGS03239			
	PaOGS02593			
Lkb1 kinase	PaOGS07226	Bger_01519	NP_001163606	serine/threonine-protein kinase 11
target of rapamycin	PaOGS00312	Bger_07577	NP_524891	target of rapamycin isoform 1
	PaOGS28942	Bger_27083		
	PaOGS16261			
Tsc1	PaOGS22671	Bger_08706	NP_477415	Tsc1
nejire	PaOGS25711	Bger_12712	NP_001188575	E1A/CREB-binding protein
	PaOGS23031	Bger_12714		
	PaOGS35225	Bger_12715		
		Bger_22304		

spargel	PaOGS23091	ND	NP_730835	PGC1
Pi3K92E	PaOGS25629	Bger_10044	NP_650902	Phosphotidylinositol 3 kinase 92E
Pi3K21B	PaOGS11039<br>PaOGS26305	Bger_15545	NP_477270	phosphatidylinositol 3-kinase 21B
Pi3K68D	PaOGS21297<br>PaOGS04183<br>PaOGS04184<br>PaOGS21298	Bger_01070	NP_001163420	Phosphotidylinositol 3 kinase 68D
Pi3K59F	PaOGS06680	Bger_06697	NP_477133	phosphotidylinositol 3 kinase 59F
protein kinase, cAMP-dependent, catalytic subunit 1	PaOGS33117	Bger_01349	NP_995672	protein kinase A
no receptor potential A	PaOGS12297<br>PaOGS02494<br>PaOGS02706	Bger_18892	NP_001284860	phospholipase C, beta
Raf	PaOGS36071	Bger_28322	NP_525047	Raf-like protein serine/threonine-protein kinase phl
Ras 85D	PaOGS07457<br>PaOGS02503	Bger_01384<br>Bger_06705	NP_476699	Ras-like protein
Ras 64B	PaOGS15449<br>PaOGS04602	Bger_00921<br>Bger_27659<br>Bger_14904	NP_523917	Ras-related protein R-Ras2
Rheb	PaOGS37079<br>PaOGS01402<br>PaOGS01401	Bger_14087	NP_730951	Ras homolog enriched in brain
RPS6-p70-protein kinase	PaOGS28537<br>PaOGS34494<br>PaOGS32255	Bger_22784<br>Bger_22785<br>Bger_27047	NP_523941	ribosomal protein S6 kinase

		Bger_27124<br>Bger_25546<br>Bger_23850		
sirtuin 1	PaOGS18856	Bger_01051<br>Bger_06322	NP_477351	NAD-dependent deacetylase sirtuin 1
sterol regulatory element binding protein	PaOGS15611	Bger_16229	NP_524166	sterol regulatory element-binding transcription factor 1
Ste20-like kinase	PaOGS15586	Bger_12288	NP_001246138	STE20-related kinase adapter protein alpha
tau	PaOGS04194	Bger_00115	NP_001303549	microtubule-associated protein tau
pollux	PaOGS20378<br>PaOGS01110	ND	NP_001163511	TBC1 domain family member 4 isoform X2<br>TBC1 domain family member 1 isoform X3<br>TBC1 domain family member 1 isoform X2
microtubule star	PaOGS27589	Bger_23717<br>Bger_25259	NP_001285725	protein phosphatase 2 (formerly 2A), catalytic subunit
twins	PaOGS22334	Bger_15088	NP_476880	phosphatase pp2a or twins
widerborst	PaOGS24441	Bger_07755	NP_733215	similar to widerborst-PC
PP2A-B'	PaOGS25786<br>PaOGS10864	Bger_00705	NP_001262695	PP2A 56 kDa regulatory subunit delta isoform
protein phosphatase V	PaOGS31508	Bger_18449	NP_001259274	serine/threonine-protein phosphatase 6 catalytic subunit
ecdysone receptor	PaOGS33228	Bger_08790	NP_001163061	Ecdysone receptor
USP	PaOGS21233<br>PaOGS36136	Bger_15477	NP_476781	ultraspiracle
withered	PaOGS29230	Bger_16466<br>Bger_16467	NP_001163112	carnitine Olmitoyltransferase 1

[1] Pame, *Periplaneta americana*, [2] Bger, *Blattella germanica*, [3] Dmel, *Drosophila melanogaster*. * = potential fragments of one gene, respectively.

Supplementary Table 21 The gene list of TOR pathway

	Pame[1] ID	Bger[2] ID	Dmel[3] ID	Supplementary notes or annotation
**Components of dTOR**				
raptor	PaOGS18513<br>PaOGS25190	Bger_12090	NP_001284924	egulatory associated protein of dTOR
rapamycin-insensitive companion of Tor	PaOGS05394	Bger_07681<br>Bger_07682	NP_573371	rapamycin-insensitive companion of dTOR
Lst8	PaOGS21452<br>PaOGS17698	Bger_13789	NP_572572	Target of rapamycin complex subunit lst8
Proline-rich Akt substrate 40 kDa	PaOGS20714	Bger_01850	NP_001303349	Proline-rich Akt substrate 40 kDa(Lobe)
target of rapamycin	PaOGS00312<br>PaOGS28942<br>PaOGS16261	Bger_07577<br>Bger_27083	NP_524891	target of rapamycin isoform 1
**Signal transduction pathway**				
4E-BP1	PaOGS17099	Bger_22986	NP_477295	Also named thor, eukaryotic translation initiation factor 4E-binding protein 1
AMPKalpha	PaOGS22353	Bger_16490	NP_477313	5'-AMP-activated protein kinase, catalytic alpha subunit
AMPKbeta	PaOGS21039	Bger_07371	NP_610460	5'-AMP-activated protein kinase, regulatory beta subunit
AMPKgama	PaOGS34091	Bger_18283	NP_001163672	5'-AMP-activated protein kinase, regulatory gamma subunit
Akt1	PaOGS21842	Bger_07332	NP_732114	serine/threonine protein kinase Akt
dishevelled	PaOGS28148	Bger_04903	NP_511118	Segment polarity protein dishevelled-like protein

				DVL-3
eIF-4E	PaOGS28859<br>PaOGS29690<br>PaOGS15419	Bger_00848<br>Bger_07664<br>Bger_00898	NP_729485	translation initiation factor 4E
Erk(rolled)	PaOGS23998<br>PaOGS05015	Bger_07900	NP_001287635	Mitogen-activated protein kinase
FK506-binding protein 2	ND	Bger_13733	NP_523792	12 kDa FK506-binding protein
pico	PaOGS35141	Bger_10233	NP_608363	Ras-associated and pleckstrin-like proteiny domains-containing protein 1
shaggy	PaOGS21420	Bger_15050<br>Bger_15051	NP_001259193	glycogen synthase kinase-3
tango	PaOGS14643	Bger_24892	NP_731308	hypoxia-inducible factor 1 beta
chico	PaOGS37252	Bger_01864	NP_723540	insulin receptor substrate-1
Lkb1 kinase	PaOGS07226	Bger_01519	NP_001163606	serine/threonine-protein kinase 11
Myc	PaOGS28552	Bger_11276	NP_525062	Myc protein
RPS6-p70-protein kinase	PaOGS28537<br>PaOGS34494<br>PaOGS32255	Bger_22784<br>Bger_22785<br>Bger_27047<br>Bger_27124<br>Bger_23850<br>Bger_25546	NP_523941	ribosomal protein S6 kinase
Phosphoinositide-dependent kinase 1	PaOGS02814	Bger_19061	NP_001261183	3-phosphoinositide-dependent protein kinase 1 isoform X1
Insulin-like receptor	PaOGS10688<br>PaOGS25540<br>PaOGS25539	Bger_10316<br>Bger_13643<br>Bger_13644	AAF55903	insulin receptor<br>similar to Insulin receptor precursor

	PaOGS31689	Bger_14014		
	PaOGS36621			
	PaOGS03239			
	PaOGS02593			
Pi3K92E	PaOGS25629	Bger_10044	NP_650902	Phosphotidylinositol 3 kinase 92E
Pi3K21B	PaOGS11039	Bger_15545	NP_477270	phosphatidylinositol 3-kinase 21B
	PaOGS26305			
Pi3K68D	PaOGS21297	Bger_01070	NP_001163420	Phosphotidylinositol 3 kinase 68D
	PaOGS04183			
	PaOGS04184			
	PaOGS21298			
Pi3K59F	PaOGS06680	Bger_06697	NP_477133	phosphotidylinositol 3 kinase 59F
inactivation no afterpotential C(PKC)	PaOGS08405	Bger_00689	NP_476863	protein kinase C
	PaOGS02145	Bger_16364		
	PaOGS04744	Bger_20742		
	PaOGS13125	Bger_27207		
Ultraspiracle（USP）	PaOGS21233	Bger_15477	NP_476781	ultraspiracle
	PaOGS36136			
Proline-rich Akt substrate 40 kDa	ND	ND	NP_001303349	
phosphatase and tensin homolog	PaOGS02078	Bger_19611	NP_599147	phosphatase and tensin-like (PTEN)
	PaOGS21433	Bger_02874		
Ras-related GTP binding A/B	PaOGS25148	Bger_02131	NP_649850	ras-related GTP-binding protein A/B
Ras-related GTP binding C/D	PaOGS00446	Bger_03026	NP_610361	ras-related GTP-binding protein C/D
Ras 85D	PaOGS07457	Bger_01384	NP_476699	Ras-like protein
	PaOGS02503	Bger_06705		

Ras 64B	PaOGS15449<br>PaOGS04602	Bger_00921<br>Bger_14904<br>Bger_27659	NP_523917	Ras-related protein R-Ras2
death related ced-3/Nedd2-like caspase	PaOGS31167	Bger_14092	NP_477249	caspase 8
Rheb	PaOGS37079<br>PaOGS01402<br>PaOGS01401	Bger_14087	NP_730951	Ras homolog enriched in brain
SAPK-interacting protein 1	PaOGS13689	Bger_14252	NP_610963	target of rapamycin complex 2 subunit
sterol regulatory element binding protein	PaOGS15611	Bger_16229	NP_524166	sterol regulatory element-binding transcription factor 1
Tsc1	PaOGS22671	Bger_08706	NP_477415	Tsc1
Mitf	PaOGS06603<br>PaOGS20366	Bger_03864	NP_001245436	microphthalmia-associated transcription factor
melted	PaOGS08596	Bger_19955	NP_001014572	melted
forkhead box, sub-group O	PaOGS31435	Bger_24290	NP_996205	similar to forkhead box subgroup O
ecdysone receptor	PaOGS33228	Bger_08790	NP_001163061	Ecdysone receptor
SCYLLA	PaOGS35498	Bger_01043	AAF59841	similar to charybde
CHARYBDE	PaOGS35498	Bger_01043	AAF59840	similar to charybde
microtubule star	PaOGS27589	Bger_23717<br>Bger_25259	NP_001285725	protein phosphatase 2 (formerly 2A), catalytic subunit
twins	PaOGS22334	Bger_15088	NP_476880	phosphatase pp2a or twins
widerborst	PaOGS24441	Bger_07755	NP_733215	similar to widerborst-PC
PP2A-B'	PaOGS25786<br>PaOGS10864	Bger_00705	NP_001262695	PP2A 56 kDa regulatory subunit delta isoform
protein phosphatase V	PaOGS31508	Bger_18449	NP_001259274	serine/threonine-protein phosphatase 6 catalytic subunit

[1] Pame, *Periplaneta americana*, [2] Bger, *Blattella germanica*, [3] Dmel, *Drosophila melanogaster*. * = potential fragments of one gene

Supplementary Table 22 Statistics of cuticle protein genes in *P. Americana* and related species

	RR-1	RR-2	RR-3	CPAPs	CPF/CPFL	TWEEDLE	Others	Totals
P. americana	30	165	18	11	10	4	1	239
B. germanica	23	74	20	10	6	1	1	135
Z. nevadensis	12	34	12	2	2	0	0	62
D. melanogaster	17	20	6	7	3	2	2	57

Supplementary Table 23 The gene list of Dpp pathway

	Pame[1] ID	Bger[2] ID	Dmel[3] ID	Supplementary notes or annotation
**Ligands and secreted factors**				
Decapentaplegic(dpp)	PaOGS32169	Bger_03413	NP_477311	Transforming growth factor-β
**Receptor**				
Thickveins(tkv)	PaOGS25735	Bger_01654	NP_787990	Transforming growth factor beta receptor, type I
Punt(put)	PaOGS32571	Bger_02266<br>Bger_02267<br>Bger_27580	NP_001262575	transforming growth factor beta receptor, type II
Saxophone(sax)	PaOGS16168	Bger_01537	NP_001246193	Transforming growth factor beta receptor, type I
**Main component**				
Mothers against dpp(mad)	PaOGS13393<br>PaOGS34042	Bger_02371<br>Bger_12546	NP_001259992	Mothers against decapentaplegic homolog
Medea(med)	PaOGS21152	Bger_05463	NP_524610	Mothers against decapentaplegic-like protein
schnurri(shn)	PaOGS24405	Bger_12964	NP_001260883	Immunodeficiency virus type I enhancer binding protein
brinker(brk)	PaOGS08043	Bger_16123	NP_511069	Brinker protein
magu	PaOGS09151	Bger_13178	NP_724871	SPARC-related modular calcium-binding protein
Daughters against dpp(dad)	PaOGS13178	Bger_02813	NP_477260	Mothers against decapentaplegic homolog
**Target genes**				
spalt major(salm)	PaOGS00024	ND	NP_723670	spalt-major-like homeotic protein
spalt-related(salr)	ND	Bger_07259	NP_523548	sal-like protein

optomotor-blind(omb)	PaOGS25538<br>PaOGS09902<br>PaOGS32020<br>PaOGS04977	Bger_01571<br>Bger_01573	NP_001259238	T-box protein

[1] Pame, *Periplaneta americana*, [2] Bger, *Blattella germanica*, [3] Dmel, *Drosophila melanogaster*.

Supplementary Table 24 The gene list of JNK pathway

	Pame[1] ID	Bger[2] ID	Dmel[3] ID	Supplementary notes or annotation
Eiger(egr)	PaOGS04629<br>PaOGS01329	ND	NP_724878	Tumour necrosis factor
Wengen(wgn)	PaOGS28432	ND	NP_728186	Tumour necrosis factor receptor
TRAF4	PaOGS29319	Bger_09414	NP_477416	TNF-receptor-associated factor 4
misshapen(msn)	PaOGS35794	Bger_13221<br>Bger_13222	NP_524679	Serine/threonine-protein kinase
TAB2	PaOGS00384	Bger_07566	NP_611408	TAK1-associated binding protein 2
TAK1	PaOGS13569	Bger_05441	NP_524080	TGF-β activated kinase 1
MKK4	PaOGS19566	Bger_17239	NP_477353	MAP kinase kinase 4
puckered(puc)	PaOGS25310	ND	NP_524273	serine/threonine protein phosphatase
Hemipterous(hep)	ND	ND	NP_727661	serine/threonine protein kinase
Basket(bsk)	PaOGS14087	Bger_26268	NP_723541	serine/threonine-protein kinase
CKA	PaOGS17383	ND	NP_001245936	Connector of kinase to AP-1
alphabet(alph)	PaOGS30503	Bger_20783	NP_651701	PPM-type phosphatase
pebbled(peb)	PaOGS01751	Bger_16145	NP_476674	transcriptional attenuator
kayak(kay)	PaOGS04497	Bger_03487	NP_001027577	AP-1 transcription factor
Jun-related antigen(jra)	ND	Bger_18999	NP_001260844	Jun-like transcription factor
anterior open(aop)	PaOGS22773	Bger_12886	NP_722766	Ets DNA-binding protein pokkuri
raw	PaOGS03631	Bger_17617	NP_723416	

[1] Pame, *Periplaneta americana*, [2] Bger, *Blattella germanica*, [3] Dmel, *Drosophila melanogaster*.

Supplementary Table 25 The gene list of GRH pathway

	Pame[1] ID	Bger[2] ID	Dmel[3] ID	Supplementary notes or annotation
Cadherin 96Ca(Cad96Ca) stit	PaOGS25866	Bger_17354	NP_651349	Tyrosine kinase receptor
	PaOGS00173	Bger_23518		
Btk family kinase at 29A(Btk29A)	PaOGS17860	Bger_14401	NP_476745	Tyrosine-protein kinase
Src oncogene at 64B(Src64B)	PaOGS22161	Bger_03956	NP_524934	Tyrosine-protein kinase
Src oncogene at 42A(Src42A)	PaOGS07754	Bger_20645	NP_610191	Tyrosine-protein kinase
	PaOGS05198			
	PaOGS31132			
grainy head(grh)	PaOGS03647	Bger_26553	NP_476845	Protein grainy head, transcription factor
	PaOGS36540			
downstream of receptor kinase(drk)	PaOGS19560	Bger_03191	NP_476858	Protein enhancer of sevenless 2B
Son of sevenless	PaOGS26097	Bger_12100	NP_476597	as GTP exchange factor, son of sevenless, putative
	PaOGS37301	Bger_16633		Ras-specific guanine nucleotide-releasing factor 2
	PaOGS05185			
rolled(rl) ERK	PaOGS23998	Bger_07900	NP_001015122	ERK, dpERK, Mitogen-activated protein kinase
	PaOGS05015			
Dopa decarboxylase(Ddc)	PaOGS13879	Bger_02884	NP_724163	Aromatic-L-amino-acid decarboxylase
	PaOGS24187	Bger_28490		
	PaOGS24186			
pale(ple)	PaOGS15510	Bger_19824	NP_476898	

[1] Pame, *Periplaneta americana*, [2] Bger, *Blattella germanica*, [3] Dmel, *Drosophila melanogaster*.

Supplementary Table 26 The gene list of Wingless pathway

	Pame[1] ID	Bger[2] ID	Dmel[3] ID	Supplementary notes or annotation
**Ligands**				
Wingless(wg)	PaOGS25726	Bger_10841	NP_523502	Wnt protein
	PaOGS35921	Bger_10842		
	PaOGS25723			
**Receptor**				
Dfz2	PaOGS10589	Bger_22240	NP_001262037	G-protein coupled receptor
arrow(arr)	PaOGS30157	Bger_02948	NP_524737	Low density lipoprotein receptor-related protein
**Main components**				
dishevelled(dsh)	PaOGS28148	Bger_04903	NP_511118	Dishevelled protein
Axin(axn)	PaOGS15378	Bger_08695	NP_733336	Axin protein
	PaOGS34949			
APC	PaOGS11938	Bger_02807	NP_477152	Adenomatous polyposis coli protein
	PaOGS18687			
pangolin(pan) TCF	PaOGS05337	Bger_21716	NP_726528	HMG-domain transcription factor
Casein kinase Iα(Ck1)	PaOGS18576	Bger_01130	NP_727631	Casein kinase I isoform alpha
	PaOGS07469			
shaggy(sgg)	PaOGS21420	Bger_15050	NP_001259193	Glycogen Synthase Kinase 3
Armadillo(arm)	PaOGS04090	Bger_25799	NP_476666	*Drosophila* homolog of beta-catenin
Groucho(gro)	PaOGS28025	Bger_00142	NP_001189294	transcription corepressor

	PaOGS08566	Bger_00145
	PaOGS31885	Bger_10352
	PaOGS06419	

[1] Pame, *Periplaneta americana*, [2] Bger, *Blattella germanica*, [3] Dmel, *Drosophila melanogaster*.

Supplementary Table 27 The gene list of Notch pathway

	Pame[1] ID	Bger[2] ID	Dmel[3] ID	Supplementary notes or annotation
**Ligands**				
Delta(Dl)	PaOGS35527	Bger_19307<br>Bger_19308	NP_477264	single pass transmembrane EGF family protein
Serrate(Ser)	PaOGS21207	ND	NP_524527	Jagged protein homolog
**Receptor**				
Notch(N)	PaOGS26746<br>PaOGS30036	Bger_03939<br>Bger_12799	NP_476859	Notch protein
**Main components**				
fringe(fng)	PaOGS08374	ND	NP_524191	
TACE	PaOGS26534<br>PaOGS26533	Bger_04995<br>Bger_02155	NP_733334	ADAM 17-like protease
dishevelled(dsh)	PaOGS28148	Bger_04903	NP_511118	Dishevelled-related protein
Numb	PaOGS17134	Bger_26118<br>Bger_21543	NP_523523	Protein numb
deltex(dx)	PaOGS02930	Bger_06392	NP_511064	RING finger domain protein
presenilin enhancer(pen-2)	PaOGS19904	Bger_19024	NP_788401	presenilin enhancer-2 subunit
Presenilin(Psn)	PaOGS37292	Bger_03812	NP_524184	Peptidase A22A
nicastrin(nct)	PaOGS15580	Bger_07196	NP_001262932	Nicastrin
anterior pharynx defective 1(aph-1)	PaOGS01429	Bger_13788<br>Bger_10399	NP_608710	Gamma-secretase subunit

mastermind(mam)	ND	Bger_01597	NP_525115	transcriptional coactivator
nejire(nej)	PaOGS25711	Bger_12712	NP_001188575	transcriptional co-activator CBP
	PaOGS23031	Bger_12714		
	PaOGS35225	Bger_12715		
Suppressor of Hairless(Su(H)) CSL	PaOGS29002	Bger_05252	NP_476868	p53-like transcription factor
Hairless(H)	PaOGS33095		NP_732533	transcription corepressor
C-terminal Binding Protein(CtBP)	PaOGS07498	Bger_25687	NP_001014617	D-isomer specific 2-hydroxyacid dehydrogenase
Groucho(gro)	PaOGS28025	Bger_00142	NP_001189294	transcription corepressor
	PaOGS08566	Bger_00145		
	PaOGS31885	Bger_10352		
	PaOGS06419			
Histone deacetylase 1(HDAC1)	PaOGS11542	Bger_19443	NP_647918	rpd3/HDA1 lysine deacetylases
	PaOGS04186	Bger_01952		

[1] Pame, *Periplaneta americana*, [2] Bger, *Blattella germanica*, [3] Dmel, *Drosophila melanogaster*.

Supplementary Table 28 The gene list of Hippo pathway

	Pame[1] ID	Bger[2] ID	Dmel[3] ID	Supplementary notes or annotation
**Signal transduction pathway**				
aPKC	PaOGS04744<br>PaOGS13125	Bger_20742<br>Bger_27610	NP_524892	atypical protein kinase C
Crumbs	PaOGS35189	Bger_00824<br>Bger_10877	NP_001247284	crb
discs large1	PaOGS13391	Bger_20914<br>Bger_20919<br>Bger_12063	NP_001259446	dlg
expanded	ND	Bger_04976	NP_476840	ex
fat	PaOGS09653<br>PaOGS17088<br>PaOGS31602<br>PaOGS35424<br>PaOGS36654<br>PaOGS23262	Bger_26078<br>Bger_26079<br>Bger_26348	NP_477497	Cadherin-related tumor suppressor
Hippo	PaOGS21447	Bger_07154	NP_611427	Serine/threonine-protein kinase
Kibra	PaOGS26283<br>PaOGS19729<br>PaOGS22053	Bger_12889	NP_001034055	protein kibra-like isoform

Lgl	PaOGS16831	Bger_12230	NP_001245804	lethal giant larvae
mob as tumor suppressor	PaOGS01717	Bger_13415	NP_001287465	mats
merlin	PaOGS19763	Bger_00850	NP_523413	Merlin
Par-1	PaOGS31633	Bger_11898	NP_995894	serine/threonine-protein kinase MARK2
	PaOGS31634	Bger_18873		
Par-6	PaOGS25037	Bger_18650	NP_573238	partitioning defective protein 6
		Bger_18651		
salvador	PaOGS04317	Bger_07756	NP_788721	Salvador-like protein 1
scribbled	PaOGS13642	Bger_22299	NP_001247321	part of the conserved machinery regulating apicobasal polarity
	PaOGS21706	Bger_22302		
	PaOGS25395			
	PaOGS35886			
warts	PaOGS32626	Bger_01745	NP_733403	tumor suppressor kinase
yorkie	PaOGS02608	Bger_18558	NP_726414	transcriptional coactivator yorkie-like isoform
	PaOGS24775	Bger_18559		

[1] Pame, *Periplaneta americana*, [2] Bger, *Blattella germanica*, [3] Dmel, *Drosophila melanogaster*.

Supplementary Table 29 The gene list of Hedgehog pathway

	Pame[1] ID	Bger[2] ID	Dmel[3] ID	Supplementary notes or annotation
**Ligands**				
Hedgehog(Hh)	PaOGS16066	Bger_02232<br>Bger_02233	NP_001034065	Hedgehog protein
**Receptor**				
Patched(ptc)	PaOGS26716<br>PaOGS26717	Bger_14050	NP_523661	Protein patched
interference hedgehog(Ihog)	PaOGS01525	Bger_23725	NP_609085	Interference hedgehog protein
smoothened(Smo)	PaOGS29029	Bger_01651	NP_523443	The seven-transmembrane protein Smoothened
**Main components**				
PKA-C1	PaOGS33117	Bger_01349	NP_723479	Protein kinase, cAMP-dependent, catalytic subunit 1
Casein kinase Iα(Ck1)	PaOGS18576<br>PaOGS07469	Bger_01130	NP_727631	Casein kinase I isoform alpha
shaggy(sgg)	PaOGS21420	Bger_15050	NP_001259193	Glycogen Synthase Kinase 3
Gprk2	PaOGS00819	Bger_20197	NP_476867	G protein-coupled receptor kinase 2
costa(cos2)	PaOGS09148	Bger_05480	NP_477092	Kinesin-like protein costa
fused(Fu)	PaOGS22218	Bger_27845	NP_477499	Serine/threonine-protein kinase
Suppressor of fused(Su(fu))	PaOGS20372	Bger_16884	NP_536750	
cubitus interruptus(Ci)	PaOGS24572<br>PaOGS01587	Bger_13404	NP_524617	Protein cubitus interruptus

[1] Pame, *Periplaneta americana*, [2] Bger, *Blattella germanica*, [3] Dmel, *Drosophila melanogaster*.

Supplementary Table 30 The list of potential growth factor genes

	Pame[1] ID	Bger[2] ID	Dmel[3] ID	Supplementary notes or annotation
**EGF**				
gurken (grk)	ND	ND	NP_476568	TGFα family signaling ligand that activates the intracellular MAP kinase pathway via the Egfr
spitz (spi)	PaOGS02437<br>PaOGS34424	Bger_24380	NP_599119	Spitz (Spi) is the cardinal Egfr ligand that is produced as a transmembrane precursor and processed by S and rho
vein (vn)	PaOGS17053	ND	NP_523942	Vein (Vn) is a secreted neuregulin-like EGFR ligand
keren (krn)	ND	ND	NP_524129	Keren is an Egfr ligand that is processed by S and rho
**FGF**				
Branchless (bnl)	PaOGS04463<br>PaOGS15926	Bger_27099	NP_732452	
Thisbe (Ths)	ND	ND	NP_610701	Thisbe (Ths) is a Fibroblast growth factor (FGF) and ligand for the FGF receptor htl
Pyramus (Pyr)	ND	ND	NP_001097275	Pyramus (Pyr) is a Fibroblast growth factor (FGF) and ligand for the FGF receptor htl
**platelet-derived growth factor and vascular endothelial growth factor**				
Pvf1	PaOGS17908<br>PaOGS32182<br>PaOGS20663<br>PaOGS37354	Bger_07155<br>Bger_08680<br>Bger_08681	NP_523407	PDGF- and VEGF-related factor 1
Pvf2	PaOGS00063	ND	NP_523499	a ligand for the Pvr receptor tyrosine kinas

	PaOGS36193			PDGF- and VEGF-related factor 2
Pvf3	PaOGS19259<br>PaOGS36193<br>PaOGS00063	Bger_15666	NP_001097107	PDGF- and VEGF-related factor 3
**TGF-b (Activin)**				
Myoglianin (Myo)	PaOGS30819	Bger_15600	NP_726604	
Activin-b (Actβ)	PaOGS19876	Bger_04898	NP_651942	
Dawdle (Daw)	PaOGS11951	Bger_01260	NP_722840	Dawdle is an Actvi- like ligand of the TGF beta superfamily. It is required for proper tricarboxylic acid cycle function in the fat body and also regulates insulin release from IPC neurons
**TGF-b (bone morphogenetic proteins (BMPs))**				
Glass-bottom boat (Gbb)	ND	Bger_02002	NP_477340	
Decapentaplegic (Dpp)	PaOGS32169	Bger_03413	NP_477311	Decapentaplegic is a ligand of the transforming growth factor-β signaling pathway that signals through Smad transcription factors
Screw (Scw)	ND	ND	NP_524863	Screw (Scw) is a BMP-type ligand that forms a heterodimer with dpp
Maverick (Mav)	PaOGS20323	Bger_02733	NP_524626	

[1] Pame, *Periplaneta americana*, [2] Bger, *Blattella germanica*, [3] Dmel, *Drosophila melanogaster*.

Supplementary Table 31 PCR primers used in this paper

**PCR primers for dsRNA.**		
gene	Forward primer	Reverse primer
Toll-1A	TAATACGACTCACTATAGGCCCCATAATTCGTCTCAAG	TAATACGACTCACTATAGGGCTGGTAGTAGTAATACAG
Toll-1B	TAATACGACTCACTATAGGGCAATACCCGACAGCGTC	TAATACGACTCACTATAGGCAATGGAACTCGTAGCG
Myd88	TAATACGACTCACTATAGGGTAGTAGGTATGGCGGGC	TAATACGACTCACTATAGGAATTCTGCTGCTGAAGCC
Dorsal	TAATACGACTCACTATAGGAAGAACGAGATGGCAACATAG	TAATACGACTCACTATAGGACCAGAAGTAAGCAGTGCCG
Akt	TAATACGACTCACTATAGGTTTCAGACTCCCAGCCGAC	TAATACGACTCACTATAGGCAGAACTCCAAGAGCCCAC
Chitin	TAATACGACTCACTATAGGCCAAGCCTACAGCGAAGGAG	TAATACGACTCACTATAGGGAGCAGCAAGCAGCAGAAAA
EcR	TAATACGACTCACTATAGGCAAAACGAGTATGAACAACC	TAATACGACTCACTATAGGGAGAAAATAACAATGGCAGT
RXR	TAATACGACTCACTATAGG ACCGCTCTGTTGAGGTTAAA	TAATACGACTCACTATAGGCAGCATCTCCATGAGGAATT
Chico	TAATACGACTCACTATAGGGACAAGACACAAAGCACAAAC	TAATACGACTCACTATAGGTCAGTGAGACACAGCA
Dsx	TAATACGACTCACTATAGGCGCCCGCTGTAGGAACCAC	TAATACGACTCACTATAGGCGCCGTCAGACAGCACTTG
InR	TAATACGACTCACTATAGGCTGCTCAAATAGCGGATGG	TAATACGACTCACTATAGGCATCTGGGCAGTTGGGAGG
Kr-h1	TAATACGACTCACTATAGGTCACCGACGCATCCACACC	TAATACGACTCACTATAGGCGAACGCTTTCCCACAG
Met	TAATACGACTCACTATAGGCACTAATGACGTTATAACGGAC	TAATACGACTCACTATAGGGACTCAGATCAGCTGGAGGTAGC
PI3K	TAATACGACTCACTATAGGCCGATGTGAGTAATGTGGC	TAATACGACTCACTATAGGTCAAGTGTGCGGTTGTAAG
Tor	TAATACGACTCACTATAGGTTACTATTTCATATCGCTGA	TAATACGACTCACTATAGGTGGACGACTATTTACACTTA
Vg	TAATACGACTCACTATAGGTTCATCAGTGAGTCGTGTGG	TAATACGACTCACTATAGGATTTGGGCTTTTTTGTTTTC
Dpp	TAATACGACTCACTATAGGAAGGACGGGCGGGAGAACTG	TAATACGACTCACTATAGGCCGACAACCGCACCCCATTA
Mad	TAATACGACTCACTATAGGTTCATACCAGGAACCCGTTT	TAATACGACTCACTATAGGAGGAGGAATCTTACAAACAG
Control	TAATACGACTCACTATAGGGAAAGCTC	TAATACGACTCACTATAGGGAATACAGCGGCCGCGAG

**Primers for qPCR**		
gene	Forward primer	Reverse primer
Toll-1A	CGACCACAATCACCTGACCA	GTCACATTGCCATGGGTTGG
Toll-1B	GCACAATGGACTGGTTGCAG	CCGCGGTTGTAGAGGAGTTT
Myd88	GTCAGTGGCGACAAGGTTCA	CTGGCATCAAGTTCCATCTCAT
Dorsal	ACACCGCGTTACAAGACACT	GGCCTACCTGAATCAAGGGG
Defensin-1	TGACCTGGCGAGCATTCAAA	CGCGGACATGTTGCCTTTTT
Defensin-2	CTTCCTGGTTTTGCTCGCAC	CTGTCCGGCTTCGGTATCTC
Defensin-3	CGTCCGCATCGCAAGAGCAA	GGCAGTGGCAGACGCCTTGTTT
Defensin-4	ACCGTGGTCCTAATCTTTCTACTGG	TCCTCCTTTGAAGCCCTTGC
Defensin-5	ACACGTCCGCATCGCAAGAT	GCCGCACAGAAGCTGTCATTAG
Attacin	TTTGATTGTTGCGACGGATTT	GCCATAGTAGTCTTTGGGTACGAGAT
Paprp-1	ATCCTAGGCCAAAGCCTTTA	TGGACTAGGGTACGGAGGAC
AFP	CTAAACTTCTGGTATTCCTTGTTCTGC	TGGTCCGTAGCTCTTGTGGG
Drosomycin	TGTGCAGTATGGGATAACGACG	CGCACCAACATTTGAGGCTAG
Termicin-1	CCTGCAAAGTTCAACTGAGT	GATTTTAAGCATTTGTGGAAT
Termicin-2	GCACAATGCCAGTTCAATAA	GCAGGTTCCGCTACTACAGA
Akt	GCAAAGAAGGGATGAAACCA	AACTCCAAGAGCCCACCAAT
Chitin	GTCATCGTGCTCAGCTCTGG	AGGTACGCTATCAGCAGGCA
EcR	ACCTGAGTACCAGTGTGCAGT	AGCTTCTCTGGCTCTGCCTT
RXR	TCTGCCAGGCGACAAACAAG	AGCACCTGGTCTGAGAGTGG
PI3K	AGTTACTGGCAACAGACGGA	CGTGAGCATTCTCGTAGCAG
Chico	CTCCTCCGAAAGTTCCACCC	TCGCAAAGAGGAACTGGGAC
Dsx	AGGAACCACCGCCTCAAGATAG	CTCCGCCGTCAGACAGCACT
InR	ATCGAAATGGAAGCAAAGGT	GGTAGGGTTGTTCAGCTAATGT
Kr-h1	GCGGCCCTACAAGTGCGATAT	CGAGTTGCCCGCTCTGTATGAA
Met	TCAGGTAAGGGTCCCGATGT	AGCTGGAGGTAGCAATTTCACA
PI3K	ACATCCCAAGAAATTCAGTTG	CCTCACGCTGGTACTTATCC
Tor	TGCACGACAAGAGCACATAA	TTGGCAGACACTTTAGACTTCA
Vg	GTGGTCATCTCAGGCACATTG	GATAAGTCCTTTCTGGCTGTTGT
Dpp	ACGTCGATGAAGACGTCAAG	CGCACGTTTGGACCTACTTA
Mad	AATGGAGGTCCTGGGAATCA	ATGCAGGAGGAGGAGTTTCT
Actin	CATCCTGCGTTTGGATCTGG	TTTCTCGTTCGGCAGTGGTG

Supplementary References

1. Terrapon, N. et al. Molecular traces of alternative social organization in a termite genome. *Nature Commun.***5**, 3636(2014)
2. Poulsen, M. et al. Complementary symbiont contributions to plant decomposition in a fungus-farming termite. *Proc. Natl Acad. Sci. USA***111**, 14500 (2014)
3. Wang, X.et al. The locust genome provides insight into swarm formation and long-distance flight. *Nature Commun.***5**, 2957(2014)
4. Harrison, M. C. et al. Hemimetabolous genomes reveal molecular basis of termite eusociality. *Nature Ecology & Evolution* (2018), in press.
5. Zhan, S., et al. The monarch butterfly genome yields insights into long-distance migration. *Cell* **147**, 1171-1185 (2011).
6. *Tribolium* Genome Sequencing Consortium. The genome of the model beetle and pest *Tribolium castaneum*. *Nature* **452**, 949-955 (2008).
7. The Honeybee Genome Sequencing Consortium. Insights into social insects from the genome of the honeybee *Apis mellifera*. *Nature* **443**, 931-949 (2006).
8. Chen, W., et al. The draft genome of whitefly *Bemisia tabaci* MEAM1, a global crop pest, provides novel insights into virus transmission, host adaptation, and insecticide resistance. *BMC Biol.* **14**, 110 (2016).
9. Mesquita, R. D., et al. Genome of *Rhodnius prolixus*, an insect vector of Chagas disease, reveals unique adaptations to hematophagy and parasite infection. *Proc. Natl Acad. Sci. USA* **112**, 14936-14941 (2015).
10. Kirkness, E. F., et al. Genome sequences of the human body louse and its primary endosymbiont provide insights into the permanent parasitic lifestyle. *Proc. Natl Acad. Sci. USA* **107**, 12168–12173 (2010).
